# Co-stimulation With TLR7 Agonist Imiquimod and Inactivated Influenza Virus Particles Promotes Mouse B Cell Activation, Differentiation, and Accelerated Antigen Specific Antibody Production

**DOI:** 10.3389/fimmu.2018.02370

**Published:** 2018-10-12

**Authors:** Can Li, Kelvin K. W. To, Anna J. X. Zhang, Andrew C. Y. Lee, Houshun Zhu, Winger W. N. Mak, Ivan F. N. Hung, Kwok-Yung Yuen

**Affiliations:** ^1^Department of Microbiology, Li Ka Shing Faculty of Medicine, University of Hong Kong, Pokfulam, Hong Kong; ^2^State Key Laboratory for Emerging Infectious Diseases, University of Hong Kong, Pokfulam, Hong Kong; ^3^Carol Yu Centre for Infection, University of Hong Kong, Pokfulam, Hong Kong; ^4^Research Centre of Infection and Immunology, University of Hong Kong, Pokfulam, Hong Kong; ^5^Department of Medicine, Li Ka Shing Faculty of Medicine, University of Hong Kong, Pokfulam, Hong Kong

**Keywords:** TLR7, imiquimod, inactivated influenza, A(H1N1)pdm09, B cell, peritoneal, mouse

## Abstract

Current influenza vaccines have relatively low effectiveness, especially against antigenically drifted strains, the effectiveness is even lower in the elderly and immunosuppressed individuals. We have previously shown in a randomized clinical trial that the topical application of a toll-like receptor 7 agonist, imiquimod, just before intradermal influenza vaccine could expedite and augment antibody response, including to antigenically-drifted strains. However, the mechanism of this vaccine and imiquimod combination approach is poorly understood. Here, we demonstrated that imiquimod alone directly activated purified mouse peritoneal B cells. When combined with inactivated H1N1/415742Md influenza virus particle (VP) as vaccine, co-stimulation of mouse peritoneal B cells *in vitro* induced stronger activation, proliferation, and production of virus-antigen specific IgM and IgG. Intraperitoneal injection of a combination of VP and imiquimod (VCI) was associated with an increased number of activated B cells with enhanced expression of CD86 in the mesenteric draining lymph nodes (mesLN) and the spleen at 18 h after injection. Three days after immunization with VCI, mouse spleen showed significantly more IgM and IgG secreting cells upon *in vitro* re-stimulation with inactivated virus, mouse sera were detected with viral neutralizing antibody. Transfer of these spleen B cells to naïve mice improved survival after lethal dose of H1N1/415742Md challenge. More importantly, the functional response of VCI-induced B cell activation was demonstrated by early challenge with a lethal dose of H1N1/415742Md influenza virus at 3 days after immunization. The spleen and mediastinal lymph nodes (mdLN) in mice immunized with VCI had germinal center formation, and significantly higher number of plasmablasts, plasma cells, and virus-antigen specific IgM and IgG secreting cells at only 3–4 days post virus challenge, compared with those of mice that have received imiquimod, inactivated virus alone or PBS. Serum virus-specific IgG2a, IgG2b, and IgG1 and bronchoalveolar lavage fluid (BALF) virus-specific IgA at 3 or 4 days post challenge were significantly higher in mice immunized with VCI, which had significantly reduced lung viral load and 100% survival. These findings suggested that imiquimod accelerates the vaccine-induced antibody production via inducing rapid differentiation of naïve B cells into antigen-specific antibody producing cells.

## Introduction

Imiquimod is a synthetic Toll-like receptor 7/8 (TLR7/TLR8) ligand that belongs to the family of chemical imidazoquinolines (1). Topical imiquimod cream has been approved by the US Food and Drug Administration for the treatment of genital and perianal warts, molluscum contagiosum, actinic keratosis and superficial basal cell carcinoma (2). The mechanism for the complete disappearance, or clearance of these warts and cancerous lesions was attributed to the stimulation of innate immune cells, such as macrophages and dendritic cells, to produce antiviral interferons (IFN-α, ⋎) and proinflammatory cytokines TNF-α, IL-6, IL-12 (2). Imiquimod also activates skin Langerhans cells, promotes their migration to reginal lymph nodes, and increases their maturation and antigen presentation activity (2). Topical imiquimod has also been studied as an adjuvant for chemotherapy or vaccination against melanoma or cutaneous metastasis (3, 4), and the mechanism is mainly associated with improved T cell response.

In recent years, imiquimod has been evaluated as a potential vaccine adjuvant (5). Our previous mouse studies showed that the combination of imiquimod with influenza vaccine induced expedited and augmented viral neutralizing antibody production (6, 7). In our previous randomized placebo controlled influenza vaccine trials, we have shown that topical imiquimod immediately followed by intradermal injection of seasonal influenza vaccine induced a robust, early and long-lasting antibody response in both young adults and elderlies (8, 9). In young adults, high titers of heterologous neutralizing antibody were also induced against antigenically-drifted seasonal influenza virus strain in addition to the vaccine virus strain (8).

The mechanism of imiquimod as a vaccine adjuvant for virus infection has been studied. For smallpox vaccine, the improvement in antibody response was associated with a Th1-biased response (10). However, it is currently unclear exactly how imiquimod improves the immunogenicity of influenza vaccine. Since the survival benefit of imiquimod adjuvant was associated with higher virus-specific antibody titers (7), it is important to delineate the role of B cells in this setting. Therefore, in this study, we specifically investigated the effect of imiquimod on B cells in the presence of influenza virus vaccine using *in vitro* and *in vivo* models.

## Materials and methods

### Animal, virus, and imiquimod

Six to eight weeks-old of female BALB/c mice obtained from Laboratory Animal Unit of the University of Hong Kong were housed in specific pathogen-free animal facility with 12 h light-dark cycle and free access to food and water. Virus challenge experiments were performed in biosafety level 2 animal laboratory. All the experimental procedures had prior approval by the Committee on the Use of Live Animals in Teaching and Research, the University of Hong Kong.

The mouse adapted A(H1N1)pdm09 strain A/415742Md/Hong Kong/2009 (H1N1/415742Md) was propagated in 10-day-old specific-pathogen-free (SPF) chicken embryos (11). Allantoic fluid was harvested and titrated on Madin-Darby canine kidney (MDCK) cells for 50% tissue culture infectious dose (TCID_50_) and plaque forming unit (PFU) (12). Fifty percent mouse lethal dose (LD_50_) was determined previously (7). To prepare inactivated virus to be used as vaccine in this study, infectious allantoic fluid was clarified by centrifugation at 1,000 rpm for 10 min, and inactivated with 0.1% formalin (v/v) at 4°C for 7 days and tested by plaque assay on MDCK cells for complete inactivation. The inactivated virus was concentrated and purified through sucrose gradient ultracentrifugation for 2 h at 28,000 rpm and 4°C. The recovered virus was re-suspended in phosphate-buffered saline (PBS) (13). HA unit and total protein concentration were determined as previously described (7).

Imiquimod (InvivoGen, USA) was dissolved with endotoxin-free water at 2.5 mg/mL and stored at −20°C in small aliquots until use.

### *In vitro* stimulation of mouse peritoneal cells

To collect whole peritoneal cells, 6 mL of cold PBS was injected into mouse peritoneal cavity and re-collected, centrifuged at 1,200 rpm for 5 min. Red blood cells were removed with RBC lysis buffer (155 mM NH_4_Cl, 12 mM NaHCO_3_, 0.1 mM EDTA). The cells were washed and counted, and cell viability was determined by trypan blue exclusion assay.

To obtain purified peritoneal B cells, peritoneal cells were first stained with a combination of fluorochrome-conjugated antibodies, anti-mouse B220-PE, anti-mouse CD11b-APC, and anti-mouse CD3-PE/Cy5 (Biolegend, USA). B cells were then sorted using a BD-FACS AriaI cell sorter (purity >95%). Both B1 cells (CD3^−^CD11b^+^B220^−/low^) and B2 cells (CD3^−^CD11b^−^B220^hi^) were harvested for subsequent studies (14, 15). The culture medium used throughout this study was RPMI-1640 complete medium containing 10% fetal bovine serum (FBS), 1% penicillin/streptomycin, 50 μM β-mercaptoethanol, 2 mM Glutamax-I, and 100 μM non-essential amino acid (NEAA; Gibco BRL). IL-4 was not included unless indicated otherwise.

To study *in vitro* activation of B cells, 2 × 10^5^/well whole peritoneal cells, or 2 × 10^5^/well purified B cells were seeded in 96-well plate in RPMI-1640 complete medium. IMQ (1 μg/mL, 2 μg/mL, or 4 μg/mL), inactivated virus (1 μg/mL, 2 μg/mL or 5 μg/mL), or a combination of IMQ (2 μg/mL) and inactivated virus (2 μg/mL) were added to the culture and incubated at 37°C, 5% CO_2_ for various time indicated in each section and collected for further analysis. In some experiments, 2 μg/ml Lipopolysaccharides (LPS, Sigma-Aldrich, Germany) were used as control.

### Immunization of mice with imiquimod and/or inactivated virus

Groups of mice were intraperitoneally injected with (a) 50 μg of imiquimod (IMQ), (b) 10 μg of inactivated H1N1/415742Md virus particle (VP), (c) a combination of IMQ (50 μg) and inactivated virus (10 μg) (VCI), or (d) PBS in a total volume of 200 μl. Peritoneal cells, spleen and mesenteric lymph nodes were collected at 18 h for analysis of peritoneal cell mobilization and B cell activation. At 3 days after immunization, spleen and serum were collected for analysis of antibody secreting cells and viral specific antibodies.

### Preparation of single cell suspension from mouse spleen and lymph nodes

Spleen and lymph node tissues were disrupted by gentle mechanical disruption and passed through 70 μm cell strainer for several times to obtain single cell suspension. Red blood cells were lysed with RBC lysis buffer. The cells were washed with RPMI-1640 complete medium. Cell viability was determined by trypan blue staining.

### Virus challenge of mice after immunization

Groups of mice received different immunization were intranasally inoculated with 10 × LD_50_ or 2 × LD_50_ doses of H1N1/415742Md virus under ketamine (100 mg/kg) and xylazine (10 mg/kg) anesthesia. At 3 days post virus challenge, spleen and mediastinal lymph nodes were taken for further assay of B cell. IgM, IgG, and IgA antibodies in serum and bronchoalveolar lavage fluid (BALF) were detected at 2, 4, and 6 days post virus challenge. Body weight and survival were monitored daily till day 14 post virus challenge.

### Spleen B cell transfer and virus challenge

Groups of mice were immunized intraperitoneally with VCI, VP, IMQ, or PBS. Spleens were collected at 3 days after immunization, spleen B cells were purified by sorting using anti-mouse CD19-FITC, and anti-mouse CD3-PE/Cy5 antibodies (purity > 95%). 5 × 10^6^ CD19^+^CD3^−^ B cells were transferred into naïve mouse by intraperitoneal injection. After 18 h of B cells transferring, the mice were intranasally inoculated with 2 × LD_50_ doses of H1N1/415742Md virus. Body weight and survival were monitored daily till day 14 post virus challenge.

### *In vitro* B cell proliferation assay

Purified peritoneal B cells 5 × 10^6^ cells/mL were labeled with 2 μM carboxyfluoresceinsuccinimidyl ester (CFSE) fluorescent dye (Thermo Fisher Scientific, USA) at 37°C for 10 min. The labeled cells were then seeded in 24-well plate (2 × 10^6^ cells/mL) in RPMI1640 complete medium and incubated at 37°C and 5% CO_2_ for 3, 4, and 5 days in the presence of appropriate stimuli. The cells were collected, and CFSE fluorescent intensities were determined by flow cytometry. The number of B cell divisions was determined by the “Proliferation Platform” using FlowJo software.

### FACS analysis

Briefly, cells were collected after different treatments and stained with combination of fluorochrome-conjugated monoclonal antibodies for cell surface markers to identify B cells and determine their activation and differentiation status. Mouse peritoneal B cells were identified by anti-B220-PE and anti-CD11b-APC as B1 cells (CD11b^+^B220^lo^) and B2 cells (CD11b^−^B220^hi^) (15, 16). Peritoneal macrophages were defined by 3-color staining with anti-I-A/I-E-Pacific Blue, anti-CD11b-APC and anti-F4/80-PE as small peritoneal macrophages (SPMs, SSC^lo^CD11b^hi^I-A^hi^F4/80^lo^), and large peritoneal macrophages (LPMs, SSC^hi^CD11b^hi^I-A^−^F4/80^hi^) (17). B cell activation was determined by staining of anti-CD19-FITC and anti-CD86-PE. Splenic and lymph node B cells were identified as CD19^+^ or B220^+^ cells (18). 4-color staining of anti-B220-PE, anti-CD11b-APC, anti-CD138-Brilliant Violet 605 and anti-GL7-Pacific Blue was used to determine B cell differentiation status. Plasma cells were identified as CD138^+^B220^−^ and plasmablasts as CD138^+^B220^+^ cells (19). Anti-CD3-PE/Cy5, anti-CD4-FITC and anti-CD8-PE were used for T cell identification. 7-Amino-Actinomycin (7-AAD) and Annexin-V-PE (BD Biosciences Pharmingen, USA) were used to detect cell apoptosis. All the antibodies used were listed in Table 1.

**Table 1:** Antibody list.

**Antibody**	**Clone**	**Supplier**
anti-mouse CD3 PE/Cy5	145-2C11	Biolegend
anti-mouse CD4 FITC	RM4-5	Biolegend
anti-mouse CD8 PE	53-6.7	Biolegend
anti-mouse B220 PE	RA3-6B2	Biolegend
anti-mouse CD11b APC	M1/70	Biolegend
anti-mouse I-A/I-E Pacific Blue	M5/114.15.2	Biolegend
anti-mouse F4/80 PE	BM8	eBioscience
anti-mouse CD19 FITC	1D3	BD Biosciences
anti-mouse CD86 PE	GL-1	Biolegend
anti-mouse CD138 Brilliant Violet 605	281-2	Biolegend
anti-mouse GL7 Pacific Blue	GL7	Biolegend
anti-mouse CD45R/B220	RA3-6B2	abcam
anti-mouse Ki-67	Rabbit Polyclonal	abcam
anti-mouse GL7	Rat Polyclonal	Thermo Fisher Scientific
anti-Rat IgG AF488	Goat Polyclonal	abcam
anti-Rabbit IgG TexasRed	Donkey Polyclonal	Jackson ImmunoResearch Laboratories
anti-mouse IgG FITC	Donkey Polyclonal	Jackson ImmunoResearch Laboratories

### Real-time RT-qPCR assay

Total RNA was extracted from 2 × 10^6^ cells stimulated *in vitro* for 1, 3, 5 days using RNeasy Mini Kit (Qiagen, USA). cDNA was synthesized using the SuperScript III system (Life Technology, USA), each target gene was amplified on the LightCycler 480 system (Roche Applied Sciences, USA) using SYBR Premix Ex Taq II system (Takara Bio Inc., Japan). The levels of *Aicda* transcripts were normalized to the level of *Cd79b*, the levels of other genes transcripts were normalized to the level of β*-actin* and then quantified by the ΔΔCT method. Primer sequences are listed in Table 2.

**Table 2:** Sequences of primers.

**Gene name**	**Forward primer (5^′^to 3^′^)**	**Reverse primer (5^′^to 3^′^)**
*β-actin*	ACGGCCAGGTCATCACTATTG	CAAGAAGGAAGGCTGGAAAAG
*IL-2*	TTCAATTGGAAGATGCTGAGA	ATCATCGAATTGGCACTCAA
*IL-4*	TTTTGAACGAGGTCACAGGA	AGCCCTACAGACGAGCTCAC
*IL-5*	AAAGAGACCTTGACACAGCTG	CCACGGACAGTTTGATTCTTC
*IL-6*	TGGAGTCACAGAAGGAGTGGCTAAG	TCTGACCACAGTGAGGAATGTCCAC
*IL-10*	CCCTTTGCTATGGTGTCCTT	TGGTTTCTCTTCCCAAGACC
*TNF-α*	ATAGCTCCCAGAAAAGCAAGC	CACCCCGAAGTTCAGTAGACA
*IFN-γ*	AAGCGTCATTGAATCACACC	CGAATCAGCAGCGACTCCTT
*Blimp1*	CCCTTTGCTATGGTGTCCTT	TGGTTTCTCTTCCCAAGACC
*Cd79b*	TGTTGGAATCTGCAAATGGA	TAGGCTTTGGGTGATCCTTG
*Aicda*	AGAAAGTCACGCTGGAGACC	CTCCTCTTCACCACGTAGCA

### Fluorescent focus microneutralization (FFMN) assay

Peritoneal B cell culture supernatant or mouse serum samples were serial 2-fold diluted and mixed with virus (M.O.I = 0.1) and incubated for 1 h at room temperature. Sample-virus mixtures were inoculated to MDCK cells in chamber slide (5 × 10^4^/well) for 1 h at 37°C. After adsorption, the cells were washed and incubated with minimum essential medium (MEM) containing 2 μg/mL of l-1-tosylamide-2-phenylethyl chloromethyl ketone (TPCK)-treated trypsin at 37°C, 5% CO_2_ for 6 h. The cells were fixed and stained with mouse anti-H1N1 influenza A nucleoprotein (NP) antibody followed by Donkey anti-mouse IgG-FITC secondary antibody (JacksonImmunoResearch, USA). The NP positive cells were examined under fluorescence microscope, and counted for 20 microscopic fields under 200 × magnification from each well using ImageJ software. Percentages of reduction of NP positive cell number were calculated against the virus only control, which was set at 100 (20, 21).

### Plaque assay and plaque reduction assay

To determine mouse lung viral load, right-side lung collected at 4 days post virus challenge were homogenized in 1 mL of cold MEM supplemented with 1% penicillin and streptomycin. The clarified supernatants were made 10-fold serial dilutions and inoculated into monolayer MDCK cells. To detect viral neutralizing antibodies in serum, samples were 2-fold serial diluted and mixed with 30 PFU of H1N1/415742Md virus. The sample-virus mixtures were incubated for 1 h at room temperature, added to MDCK cells. After virus adsorption for 1 h at 37°C, the cells were overlaid with MEM containing 2 μg/mL of TPCK-treated trypsin and 2% low-melting agarose, and further incubated at 37°C for 72 h. After staining with 0.5% crystal violet, the numbers of plaques were counted. Uninfected mouse serum samples were used as negative controls. Percentages of plaque reduction in tested samples were calculated against the negative control, which was set at 100.

### Enzyme-linked immunospot (ELISPOT) assay

To determine IgM or IgG secreting cells, goat anti-mouse IgM or IgG (Biolegend, USA) were coated to 96-well filtration plates (Millipore) at 4°C for overnight. For detection of virus-specific IgM or IgG producing cells, 100 μL of purified inactivated H1N1/415742Md virus (5 μg/mL) were coated. Pre-stimulated cells were transferred to coated plate (2 × 10^5^ cells/well) and further incubated at 37°C, 5% CO_2_ for 6 h for total IgM, IgG detection, and 24 h for virus-specific IgM and IgG. Alkaline phosphatase (AP) conjugated-goat anti-mouse IgM or IgG antibody (Invitrogen, USA) was added and incubated for 2 h at room temperature, then reacted with BCIP/NBT substrate (Sigma-Aldrich, USA) for 5 min. The spots were counted by CTL ImmunoSpot reader (Cellular Technology Ltd., USA).

### Enzyme-linked immunosorbent assay (ELISA)

For detection of virus specific antibodies in mouse serum and BALF, 96-well immunoplates (Nunc-Immuno Modules; Nunc A/S, Denmark) were coated with inactivated H1N1/415742Md virus (2 μg/mL). 2-fold serially diluted serum or BALF was tested in duplicate. The optical density (OD) was read at 450 nm. The cut-off OD was set at the mean OD of uninfected samples at all dilutions plus 3 standard deviations. The highest sample dilution which produces an OD above this cut-off OD was taken as the antibody titer (7).

### Immunofluorescent staining of germinal center in mouse spleen

Mouse spleens were collected at 4 days post challenge and processed to frozen tissues sections. After rehydration in PBS and permeabilized with 0.25% Triton X-100 in PBS, the sections were blocked with 1% PBS/BSA and then with 0.1% Sudan Black B to reduce autofluorescence. The slides were stained with primary antibodies (anti-mouse CD45R/B220 and anti-mouse Ki-67, or anti-mouse CD45R/B220 and anti-mouse GL7) at 4°C overnight followed by fluorochrome-conjugated secondary antibodies. Stained sections were examined using Olympus BX53 microscope. Images were captured with digital camera and processed with Olympus cellSens Dimension 1.17 software.

### Statistical analysis

Mouse survival rates after virus challenge in different groups were analyzed by the Kaplan-Meier method and the log rank test. Serum antibody titers were compared by the Mann-Whitney U test. One-way ANOVA with Tukey's *post-hoc* test was used for analysis and comparison between different groups. A value of *P* < 0.05 was considered statistically significant.

## Results

### VCI stimulated peritoneal B cells to produce higher levels of antibodies with viral neutralizing function *in vitro*

**Figure 1:**
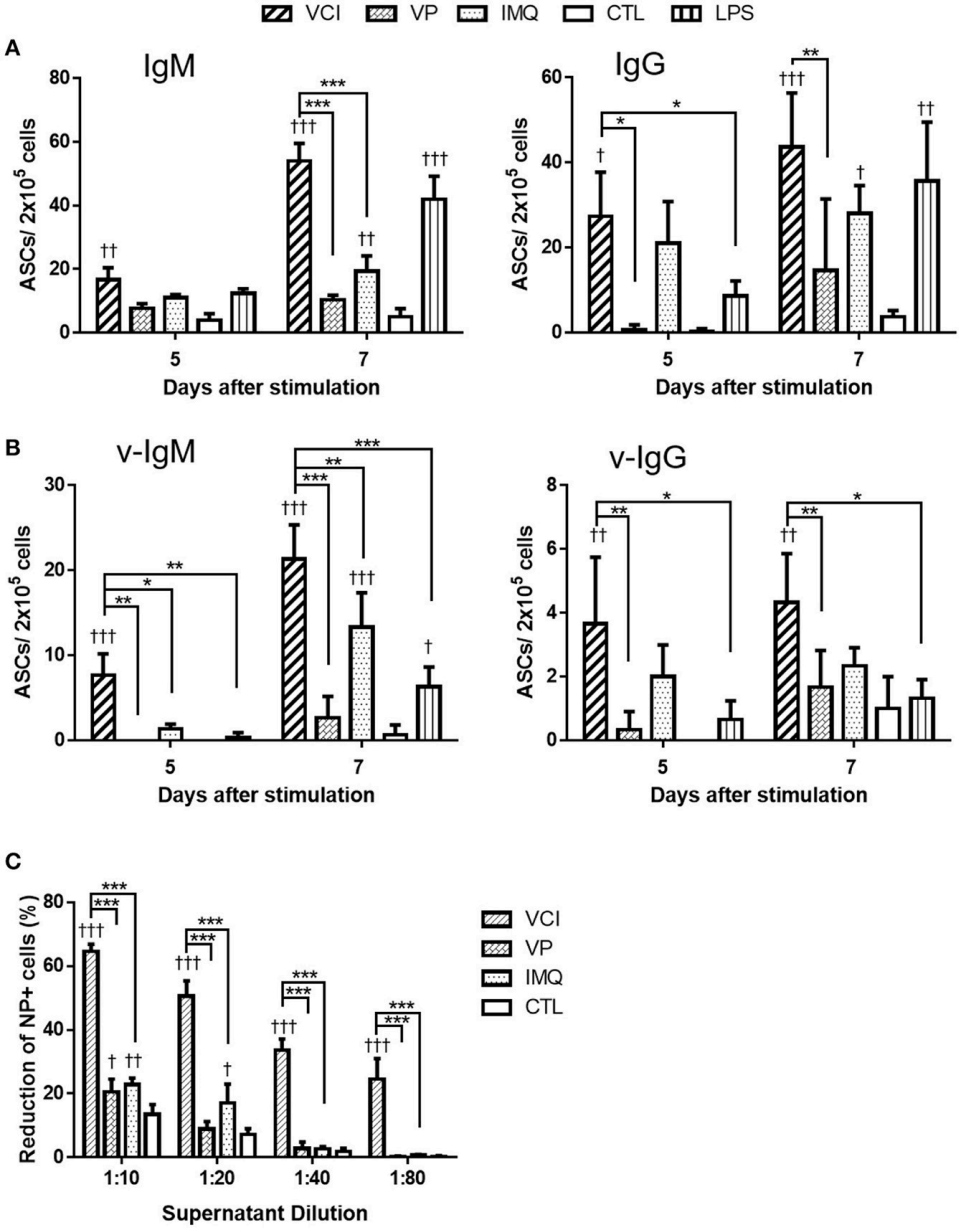
Antibody secretion in peritoneal B cells after *in vitro* stimulation. Purified mouse peritoneal B cells were cultured in RPMI 1640 complete medium containing VCI (IMQ 2 μg/mL + VP 2 μg/mL), VP 2 μg/mL, IMQ 2 μg/mL, LPS 2 μg/mL, or medium only (CTL). At 5 or 7 days after stimulation, the culture supernatant were collected for FFMN assay, the cells were transferred to ELISPOT assay plates coated with anti-mouse IgM, anti-mouse IgG or inactivated H1N1/415742Md virus, and further incubated for 24 h for detection of IgM or IgG secreting cells. **(A)** Number of total IgM (left) or IgG (right) secreting cells detected by ELISPOT. **(B)** Number of viral specific IgM (v-IgM, left) or IgG (v-IgG, right) secreting cells detected by ELISPOT. Data presented are mean of three different experiments. Error bar indicated standard deviation. *n* = 3 ^†^*p* < 0.05; ^†^^†^*p* < 0.01; ^†^^†^^†^*p* < 0.001 (compared with CTL group). ^*^p < 0.05; ^**^p < 0.01; ^***^p < 0.001 (comparing between VCI group with other treatment group). **(C)** Viral neutralizing antibody in B cell culture supernatant at 7 days after stimulation, determined by FFMN assay. MDCK cells were inoculated with 0.1 M.O.I. of H1N1/415742Md virus mixed with or without above mentioned B cell culture supernatant at different dilution and incubated for 6 h, the infected cells were stained for viral nucleoprotein (NP). Percentage of reduction of the number of NP positive cells calculated against virus only infected MDCK cells. Error bar indicates standard deviation. *n* = 3 repeated tests. ^†^*p* < 0.05; ^†^^†^*p* < 0.01; ^†^^†^^†^*p* < 0.001 (compared with CTL group). ^***^p < 0.001 (compared between different groups).

We have previously shown that intraperitoneal VCI immunized mice had higher serum antibody titers than VP immunized mice (6). To determine whether the enhanced antibody response is due to the direct effect of VCI on peritoneal B cells, we stimulated peritoneal B cells from naïve mice with VCI, VP, IMQ, LPS or medium only *ex vivo* for 5 or 7 days and determined antibody secreting B cells (ASC) by ELISPOT. We found that VCI, VP, or IMQ all increased the number of IgM (Figure 1A, left) or IgG (Figure 1A, right) secreting cells at 5 days post stimulation, and further increased significantly at 7 days post stimulation when compared with medium control. VCI stimulation induced significantly higher number of both IgM and IgG secreting B cells comparing with VP stimulation. IMQ alone increased IgM or IgG secreting B cells, but lower than the number in VCI group. The numbers of B cells secreting viral specific IgM (v-IgM) or IgG (v-IgG) were also significantly higher in the VCI group than VP group (Figure 1B). Whereas v-IgG induced by VCI did not reach statistical significance comparing with IMQ alone (Figure 1B). Representative ELISPOT images for these tests were shown in Supplementary Figure S1.

Determination of viral neutralizing antibody in the culture supernatant by FFMN assay showed that the peritoneal B cell culture supernatant after 7 days of stimulation with VCI significantly reduced the number of MDCK cells expressing viral nucleoprotein by 55% at 1:20 dilution, 33% at 1:40 dilution at 6 h post virus inoculation, which were significantly higher than that of VP or IMQ stimulated cells (Figure 1C, and Supplementary Figure S2). These results indicated that VCI activated and directed peritoneal B cells toward differentiation into functional antibody production more effectively than VP alone.

### VCI directly induced mouse peritoneal B cells activation and proliferation with reduced B cell apoptosis *in vitro*

**Figure 2:**
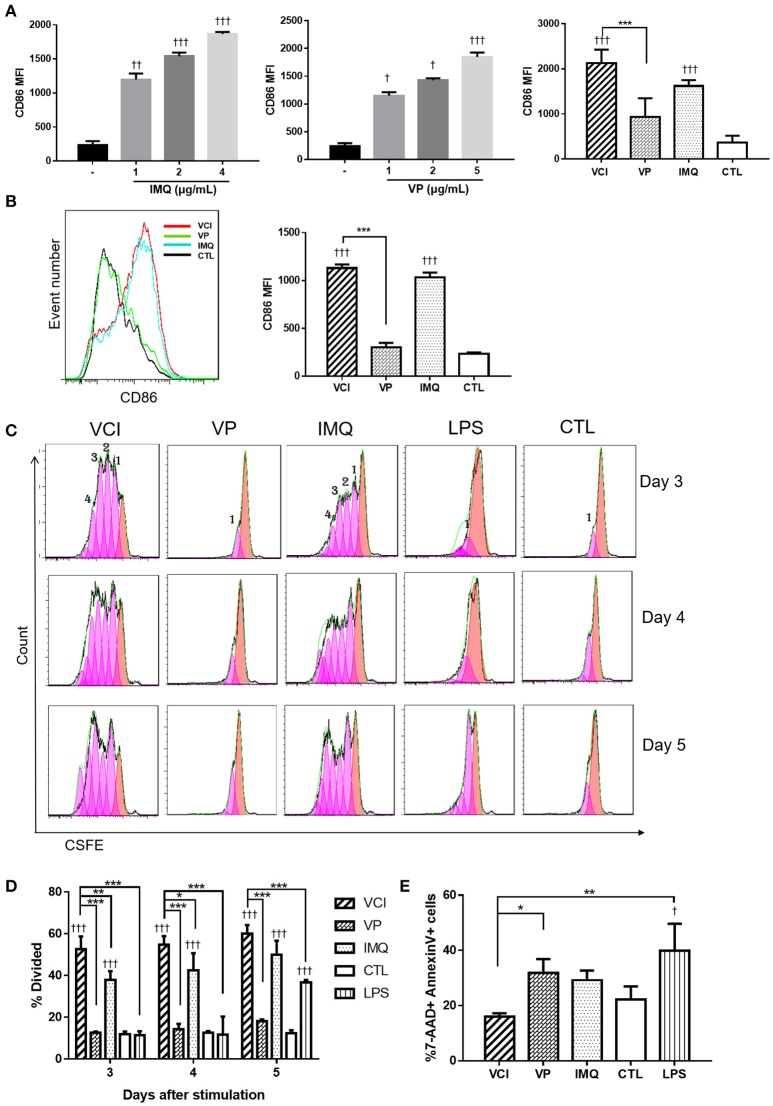
*In vitro* activation, proliferation and apoptosis of mouse peritoneal B cells induced by different stimulations. **(A)** Whole peritoneal cells were cultured in RPMI1640 complete medium with or without IMQ, VP, or VCI for 24 h. The cells were then stained with FITC-CD19 (B cell marker) and PE-CD86 (B cell activation marker) antibodies. Mean fluorescent intensity (MFI) of CD86 expression on B cells stimulated with different doses of IMQ (left panel), different doses of VP (middle panel) or VCI (IMQ 2 μg/mL + VP 2 μg/mL), VP 2 μg/mL, IMQ 2 μg/mL, and medium control (CTL; right panel). **(B)** Purified peritoneal B cell activation by VCI (IMQ 2 μg/mL + VP 2 μg/mL), VP (2 μg/mL), IMQ (2 μg/mL), or medium control. CD86 expression histogram (left panel) and MFI of CD 86 (right panel) after 24 h stimulation. **(C)** Peritoneal B cells *in vitro* proliferation determined by flow cytometry assay. Representative histogram of CFSE labeling profiles of purified peritoneal B cells cultured in the presence of VCI, VP, IMQ as above, and 2 μg/mL LPS or CTL for 3, 4, or 5 days. The numbers indicate generations of cell division. **(D)** Percentage of cells that has undergone division induced by different stimulations. **(E)** B cell apoptosis determined by flow cytometry. Purified peritoneal B cells were stimulated as above for 3 days and stained by Annexin V and 7-AAD. Percentage of 7-AAD^+^/Annexin V^+^ apoptotic B cells showed in bar chart. Data are represented as mean value of three different experiments. Data presented are mean of two different experiments. Error bar indicates standard deviation. *n* = 3–6 for each group. ^†^*p* < 0.05; ^†^^†^*p* < 0.01; ^†^^†^^†^*p* < 0.001 (compared with medium control). ^*^p < 0.05; ^**^p < 0.01; ^***^p < 0.001 (compared between different groups).

To prove VCI has potent action on peritoneal B cells, we further characterized VCI stimulated peritoneal B cells. Firstly, whole peritoneal cells isolated from naïve BALB/c mice were incubated with various concentrations of IMQ, or VP alone, VCI, or PBS for 24 h. Flow cytometry analysis showed that both IMQ and VP dose-dependently increased the expression of B cell activation marker CD86 (CD19^+^CD86^+^; Figure 2A, left & middle). However, VCI combination induced significantly higher level of CD86 expression when compared with VP alone, but not statistically higher than IMQ alone (Figure 2A, right; Supplementary Figure S3). This suggested although VP alone can stimulate B cell activation in the presence of other antigen presenting cells, IMQ further enhanced the VP-induced peritoneal B cell activation.

Cells isolated from the mouse peritoneal cavity consist of numerous immune cells, including B cells (~50%), macrophages (~40%), neutrophils, eosinophils and mast cells (17, 22). The interplay between B cells and other immune cells may affect B cell activation. To determine whether VCI, VP, or IMQ has direct effect on B cells, we performed the experiment with purified peritoneal B cells. As shown in Figure 2B, VCI and IMQ induced significantly higher levels of CD86 expression than that of VP. VP did not increase the expression of CD86 on purified B cells comparing with control group (Figure 2B). There was also no significant difference in CD86 expression between VCI and IMQ stimulated B cells. These data suggested that IMQ directly activated peritoneal B cells, which did not require the presence of and activation by other peritoneal immune cells. On the other hand, VP alone could not sufficiently activate purified B cells and required help from other peritoneal immune cells.

To study the effect of VCI on B cell proliferation, CSFE fluorescent dye labeled purified peritoneal B cells were treated with VCI, VP, or IMQ for 3–5 days. LPS was included as a control. VCI and IMQ induced 4 generations of B cell division at 3 days post stimulation, while VP alone did not induce any cell proliferation even up to day 5 post stimulation (Figure 2C). The percentage of divided cells induced by VCI were significantly higher when compared with that of VP, IMQ or control group (Figure 2D).

Since *in vitro* B cell activation will also trigger cell apoptosis, we determined the percentage of apoptotic cells at 3 days post-stimulation using flow cytometry assay. The percentage of 7-AAD^+^Annexin V^+^ was significantly lower in VCI group (16.4%) than that of VP alone (31.8 %) or IMQ alone (29.2%) groups (Figure 2E). This suggested that B cells stimulated by VCI had longer survival when compared with cells treated with IMQ or VP.

**Figure 3:**
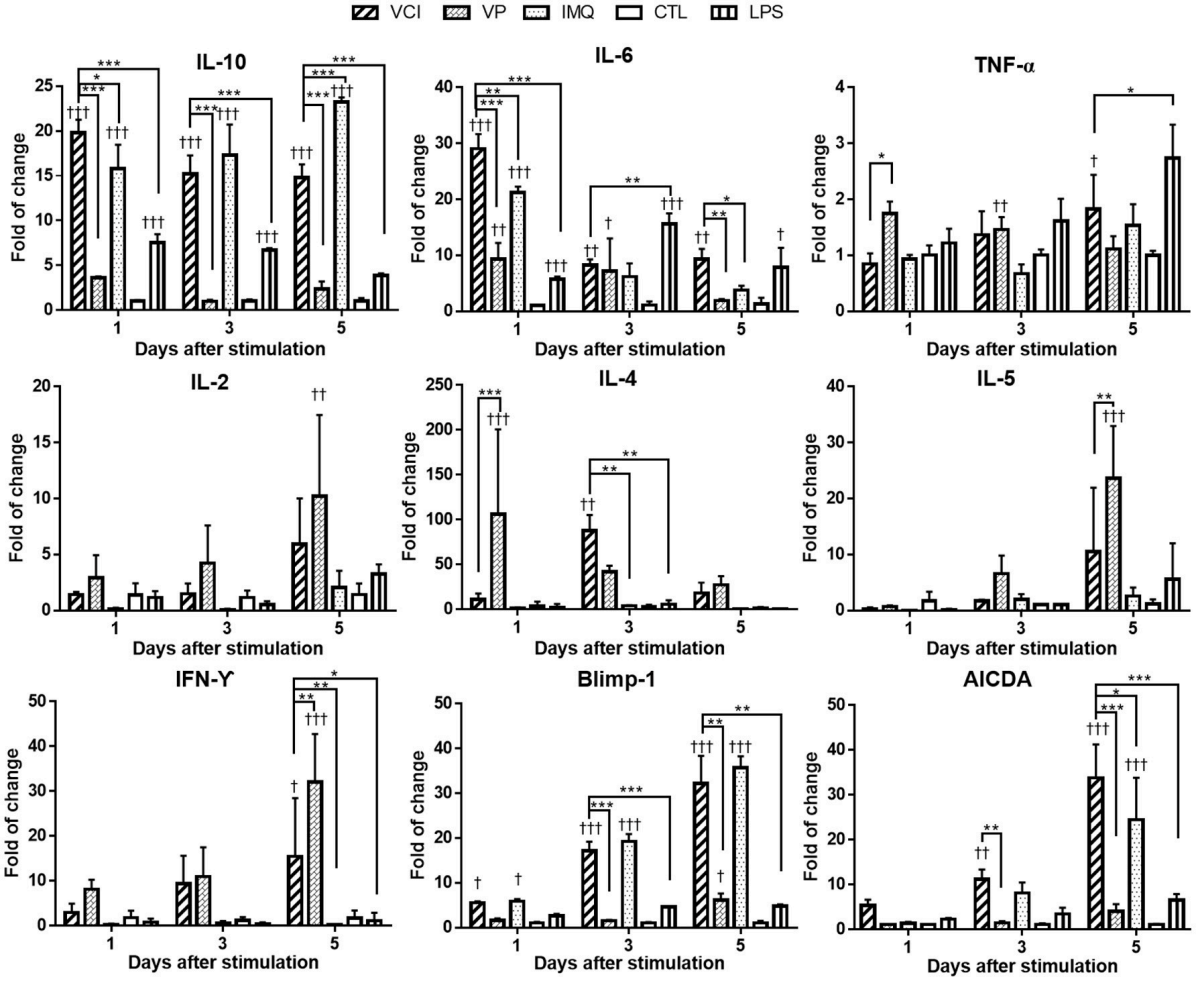
Cytokines and B cell differentiation genes expression in mouse peritoneal B cells after *in vitro* stimulation. Purified peritoneal B cells were stimulated with VCI (IMQ 2 μg/mL + VP 2 μg/mL), VP 2 μg/mL, IMQ 2 μg/mL, LPS 2 μg/mL or medium control (CTL) for 1, 3 and 5 days. Cells were collected for RNA extraction and real time RT-PCR determination of gene expression. Fold change relative to the unstimulated purified peritoneal B cells. Data presented are mean of three different experiments. Error bar indicates standard deviation. n = 6. ^†^*p* < 0.05; ^††^*p* < 0.01; ^†^^†^^†^*p* < 0.001 (compared with medium control). ^*^p < 0.05; ^**^p < 0.01; ^***^p < 0.001 (compared between different groups).

Cytokine gene responses in these peritoneal B cells were studied by real time RT-qPCR. The results showed different pattern and magnitude of cytokine gene expressions in VCI, VP, or IMQ stimulated peritoneal B cells. IL-10 was significantly upregulated after VCI or IMQ stimulation comparing with that of VP (Figure 3); VP alone only slightly upregulated the expression of IL-10. IL-6 expression was increased dramatically by VCI or IMQ stimulation at 1 day, but decreased to lower level at 3 days and 5 days. The expression of TNF-α was not much induced in B cells in all the stimulation groups (Figure 3). VP induced higher levels of IL-2, IL-4, IL-5, and IFN-γ comparing with that of VCI, while IMQ alone did not increase the expression of these cytokine genes. Study of B cell differentiation related genes Blimp-1 and AICDA expression showed that VCI and IMQ alone upregulated significantly the expression of these two genes, continued increasing from 1 to 5 days post stimulation. The differences between VCI and IMQ stimulated B cells did not reach statistical significance at most of the time points (Figure 3). However, VP stimulation only induced constantly lower level of Blimp-1 and AICDA expression from 1 to 5 days post stimulation, which were statistically significant lower than that of VCI stimulated B cells (Figure 3).

### *In vivo* administration of VCI changed peritoneal cell populations and increased spleen and mesenteric lymph node B cell number and activation

**Figure 4:**
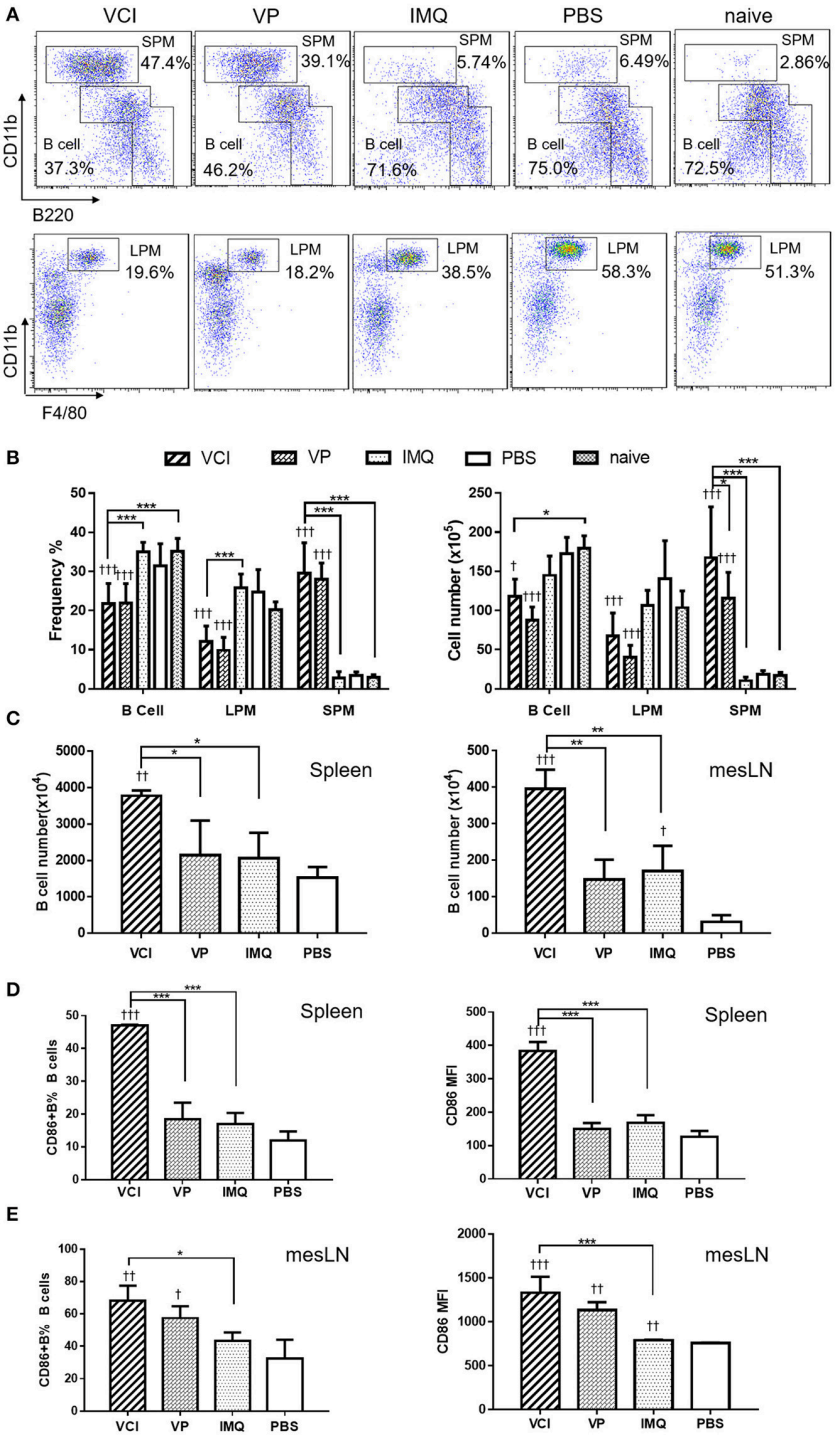
Mouse peritoneal cell mobilization and B cell activation after intraperitoneal administration of VCI (IMQ 50 μg + VP 10 μg), VP 10 μg, IMQ 50 μg in 200 μl, or 200 μl of PBS. **(A)** Representative flow cytometry profile of mouse peritoneal cells obtained 18 h after i.p. injection. **(B)** Percentage (left) and absolute cell number (right) of peritoneal B cells (B1+B2 cells), large macrophage (LPM) and small macrophage (SPM). Data presented are mean of three different experiments. **(C)** Intraperitoneal administration VCI activated B cells in spleen and mesenteric lymph node (mesLN). Eighteen hours after i.p. injection of VCI, IMQ, VP or PBS as above, spleen and mesLN were analyzed. The cell numbers were counted and the cells were stained with FITC-CD19 and PE-CD86 antibodies for flow cytometry assay. B cell number = total cell number × B cell percentage determined by flow cytometry in spleen and mesLN. **(D)** Percentage of CD86 expressing B cells (left) and MFI of CD86 (right) in spleen. (**E**) Percentage of CD86 expressing B cells (left) and MFI of CD86 (right) in mesLN. Data presented are mean of two experiments. Error bar indicates standard deviation. *n* = 4–6. ^†^*p* < 0.05; ^††^*p* < 0.01; ^†*††*^*p* < 0.001 (compared with PBS group). ^*^p < 0.05; ^**^p < 0.01; ^***^p < 0.001; (comparing between VCI group with other treatment group).

To study how VCI combination induces B cell responses *in vivo*, naïve mice were intraperitoneally injected with VCI, IMQ, VP, or PBS. Peritoneal cells were analyzed at 18 h post injection. The frequency and absolute number of peritoneal B cells were significantly reduced in mice received VCI or VP injection comparing with those of PBS-treated mice, untreated naïve mice, and mice treated with IMQ (Figures 4A,B). Large peritoneal macrophages (LPM, CD11b^hi^F480^+^B220^−^) were also significantly reduced after VCI or VP treatment (Figure 4B). On the contrary, the frequency and number of small peritoneal macrophages (SPM, CD11b^hi^B220^−^) increased significantly in mice treated by VCI and VP (Figure 4B). There was no difference in the number of B cells, LPM and SPM between VCI and VP groups. These data suggest that intraperitoneally injected VCI or VP has markedly affected different populations of peritoneal cells, which may involve the mobilization of peritoneal B cells and large macrophages out of the peritoneal cavity, while a large number of small macrophages were recruited.

To study whether intraperitoneal VCI immunization promotes B cell responses in mouse spleen and draining lymph nodes, spleens and mesenteric lymph nodes (mesLN) were analyzed at 18 h after intraperitoneal injection. Comparing with VP, IMQ, or PBS, mice immunized with VCI had a significantly higher number of B cells in the spleen and mesLN (Figure 4C). VCI immunized mice also had significantly higher frequency of CD86 expressing B cells and significantly higher expression level of CD86 on those splenic B cells comparing with that of VP and IMQ immunized mice (Figure 4D). In the mesLN, the level of CD86 expression on B cells was significantly higher in VCI-immunized mice than those of IMQ alone or PBS treated mice (Figure 4E). These data indicated that intraperitoneal immunization with VCI potently and rapidly elicited B cell activation in the spleen and mesLN.

### *In vivo* administration of VCI induced antibody response in spleen

**Figure 5:**
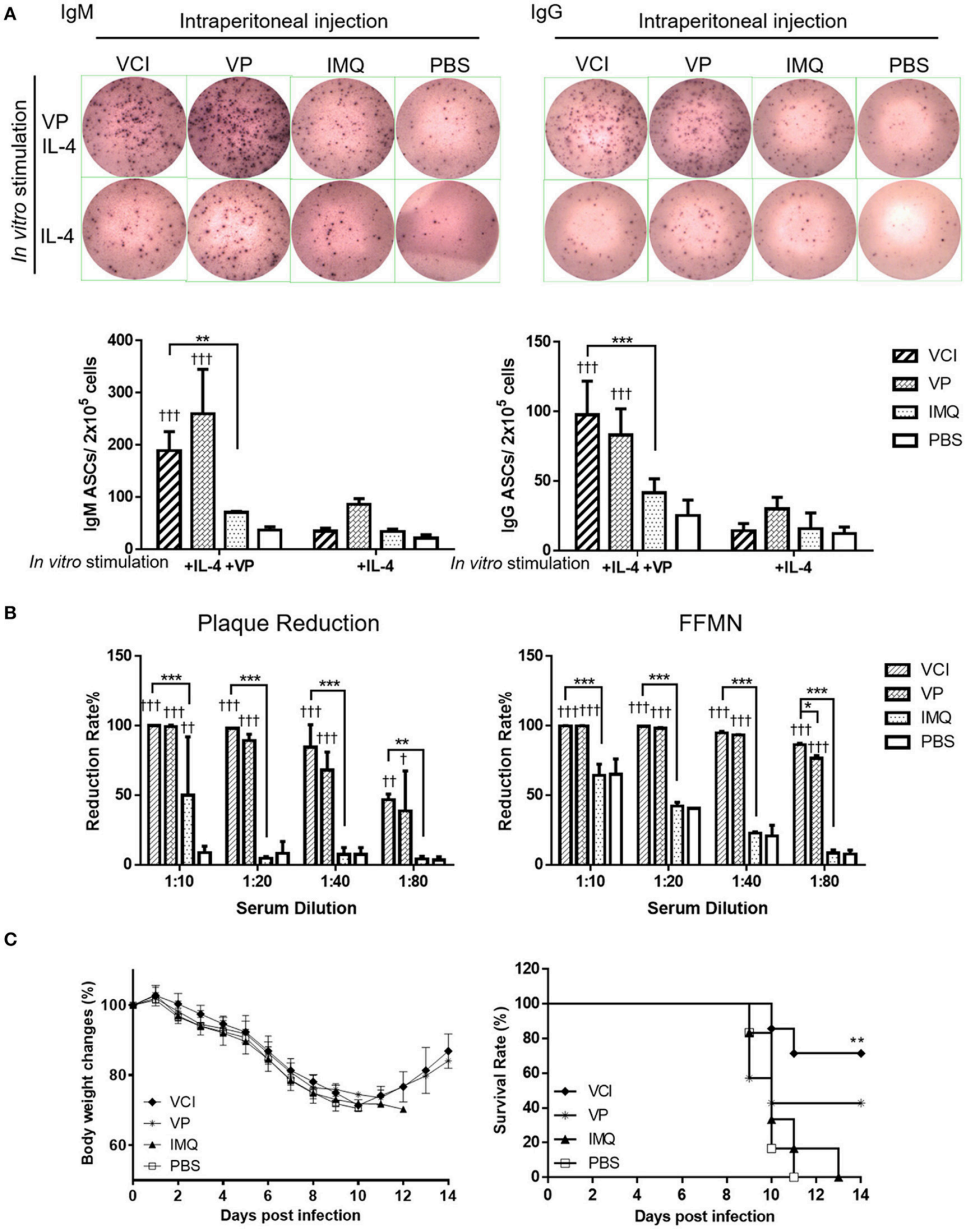
Antibody producing B cells in mouse spleen after immunization. Mice received intraperitoneal administration of VCI (IMQ 50 μg + VP 10 μg), IMQ (50 μg), VP (10 μg), or PBS. Spleen single cell suspensions were prepared at 3 days after immunization and cultured in the presence of 3 ng/mL of recombinant mouse IL-4 and with or without 2 μg/mL inactivated H1N1/415742Md virus. After 48 h of culture, cells were transferred into ELISPOT filter plates. **(A)** Representative ELISPOT images for IgM and IgG secreting cells (upper panel); and the number of IgM (left) and IgG (right) secreting cells (lower panel). *n* = 7. **(B)** Virus neutralizing antibody determined by plaque reduction and FFMN assays. Mouse sera were collected at 3 days after immunization as above mentioned and tested for viral neutralizing antibody. Percentage of plaque reduction calculated against virus only (left); and percentage of reduction of the NP+ cell numbers in FFMN assay (right). Error bar indicates standard deviation. n = 3. ^†^*p* < 0.05; ^†^^†^*p* < 0.01; ^†^^†^^†^*p* < 0.001 (compared with PBS group). ^*^p < 0.05; ^**^p < 0.01; ^***^p < 0.001; (compared between VCI group with other treatment group). **(C)** Body weight change (left) and survival (right) of mice transferred with immunized mice spleen B cells against H1N1/415742Md virus challenge. After 3 days of immunization as above, 5 × 10^6^ spleen B cells were purified from immunized mice and transferred into naïve mice by intraperitoneal injection. After 18 h of B cells transfer, the mice were intranasally inoculated with 2 × LD_50_ doses of H1N1/415742Md virus. Body weight and survival were monitored for 14 days. Data presented are mean of two experiments, *n* = 6–7. ^**^p < 0.01 comparing with the survival of PBS group.

To study whether the spleen B cells activation by intraperitoneal immunization of VCI would develop into antibody production. First, we studied how the splenocytes respond to viral antigen stimulation *in vitro*. At 3 days after intraperitoneal immunization as above, splenocytes were stimulated *in vitro* with 2 μg/mL of inactivated H1N1/415742Md virus for 48 h. In this experiment, IL-4 stimulation without viral antigen was used as control. ELISPOT showed that significantly more IgM, IgG producing cells were elicited by viral antigen stimulation of splenocytes from VCI-immunized mice when compared with those splenocytes from mice immunized with IMQ or PBS (Figure 5A). The splenocytes of mice treated with VP produced even higher frequency of IgM producing cells than VCI, but did not reach statistically significance. As expected, all mice had IgM, IgG secreting cells responses, but to a much less degree compared with those cells stimulated with viral antigen (Figure 5A), which indicated VCI stimulated responses against viral antigen.

Meanwhile, to show viral neutralizing antibody production *in vivo*, serum samples were tested concurrently after 3 days intraperitoneally immunization with VCI, VP, IMQ or PBS control. Plaque reduction assay showed that both VCI and VP induced early viral neutralizing antibody against H1N1/415742Md virus, which reduced the number of plaque by 50% at 1:80 dilution, but no plaque reduction activity was detected in IMQ or PBS immunized mouse sera even at 1:20 dilution (Figure 5B left, Supplementary Figure S4A). However, there was no statistically significant difference between VCI and VP immunized sera. FFMN assay also showed the same results (Figure 5B right, Supplementary Figure S4B).

We then further investigated whether these spleen B cells confer any degree of protection to mice when challenged with homologous influenza virus. 5 × 10^6^ purified spleen B cells isolated from mice after 3 days intraperitoneally immunized with VCI, VP, IMQ, or PBS were transferred to groups of naïve mice by intraperitoneal injection, the mice were then challenged with 2 × LD_50_ of H1N1/415742Md virus 18 h later. The results showed that mice received spleen B cells from VCI immunized mice had 71.4% survival, whereas the mice received IMQ immunized B cells or PBS control B cells had 100% mortality. VP immunized mouse spleen B cells transfer also conferred 42.8% survival to infected recipient mice (Figure 5C). These results indicated that VCI and VP activated B cells can provide protective immunity to some degree though there was not much improvement of body weight loss (Figure 5C).

### *In vivo* administration of VCI activated B cells in mouse spleen and lymph node differentiated rapidly in response to early virus challenge 3 days post immunization

**Figure 6:**
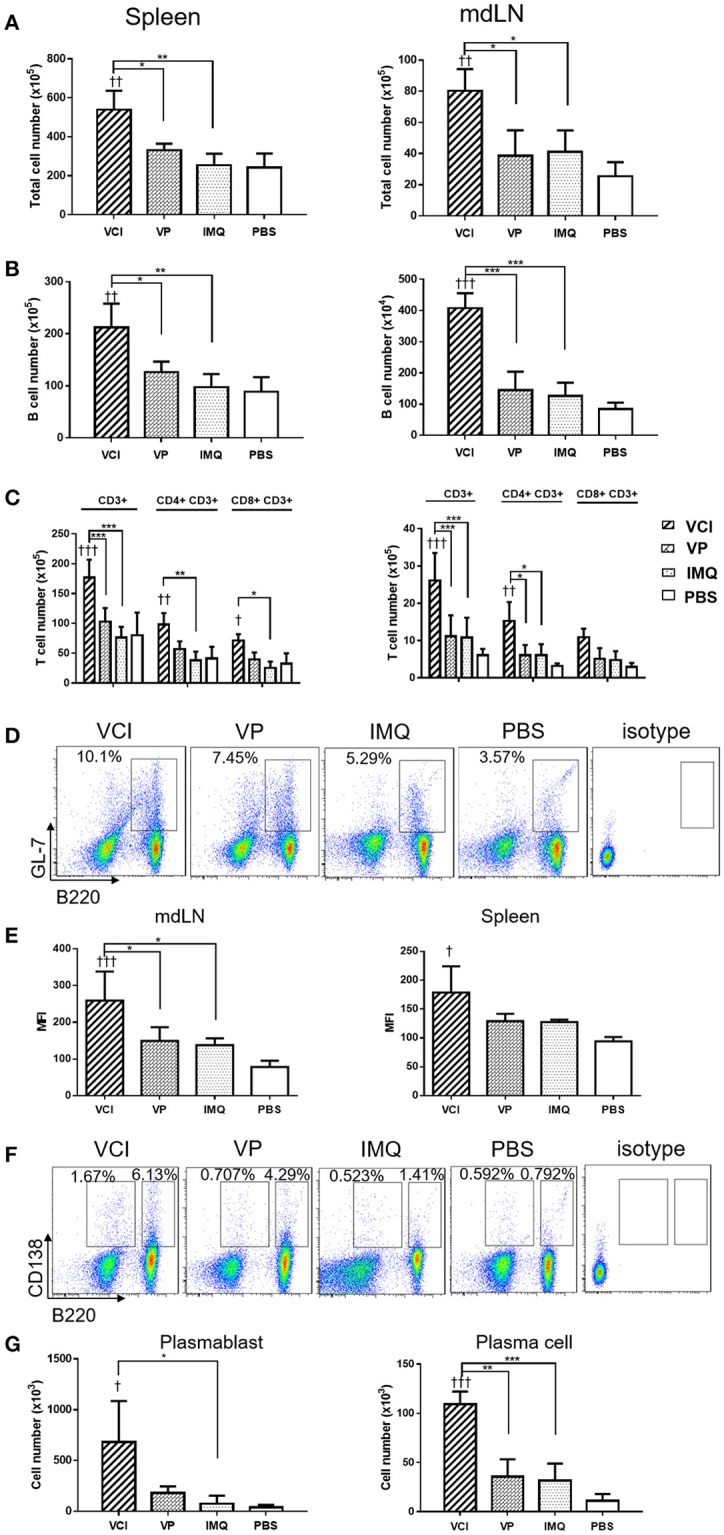
B cell responses in VCI immunized mice at 3 days after H1N1/415742Md virus challenge. Mice received intraperitoneal administration of VCI (IMQ 50 μg + VP 10 μg), IMQ (50 μg), VP (10 μg), or PBS for 3 days, and then were challenged with 10 × LD_50_ of H1N1/415742Md virus intranasally. At 3 days post virus challenge, spleens and mdLNs were examined. **(A)** Increased total cell count of spleen (left) and mdLN (right). **(B)** Total B cells numbers in the spleen (left) and mdLN (right) determined by flow cytometry. **(C)** T cells numbers in the spleen (left) and mdLN (right) determined by flow cytometry. **(D)** Representative flow cytometry dot plot of GL7 expression on B cells in spleen. **(E)** MFI of GL7 expression on B cells in mdLN (left) and spleen (right). **(F)** Representative flow cytometry dot plot of plasmablast (B220^+^CD138^+^), and plasma cell (B220^−^CD138^+^) in the mdLN, cell gated on lymphocyte singlets. **(G)** Plasmablasts (left) and plasma cells (right) number detected in mdLN. *n* = 3 for all experiments. ^†^*p* < 0.05; ^†^^†^*p* < 0.01; ^†^^†^^†^*p* < 0.001 (compared with PBS group). ^*^p < 0.05; ^**^p < 0.01; ^***^p < 0.001; (compared VCI group with other treatment group).

To provide more evidences that VCI immunization induces rapidly functional differentiation of B cells upon encountering influenza virus *in vivo*, mice received intraperitoneal injection of VCI, VP, IMQ or PBS for 3 days were challenged with 10 × LD_50_ of H1N1/415742Md virus intranasally. At day 3 post virus challenge, spleen and mediastinal lymph nodes (mdLN) were taken for analysis. First, we found increased cellularity in VCI immunized mouse spleen and mdLN. As shown in Figures 6A,B, significantly higher number of total cells and B cells were detected in the spleen and mdLN of VCI immunized mice comparing with those mice immunized with VP, IMQ alone, or PBS. Total T cells, CD4^+^ and CD8^+^ T cells were also increased significantly in the spleen of the VCI-immunized mice when compared to the mice immunized by VP, IMQ or PBS (Figure 6C).

**Figure 7:**
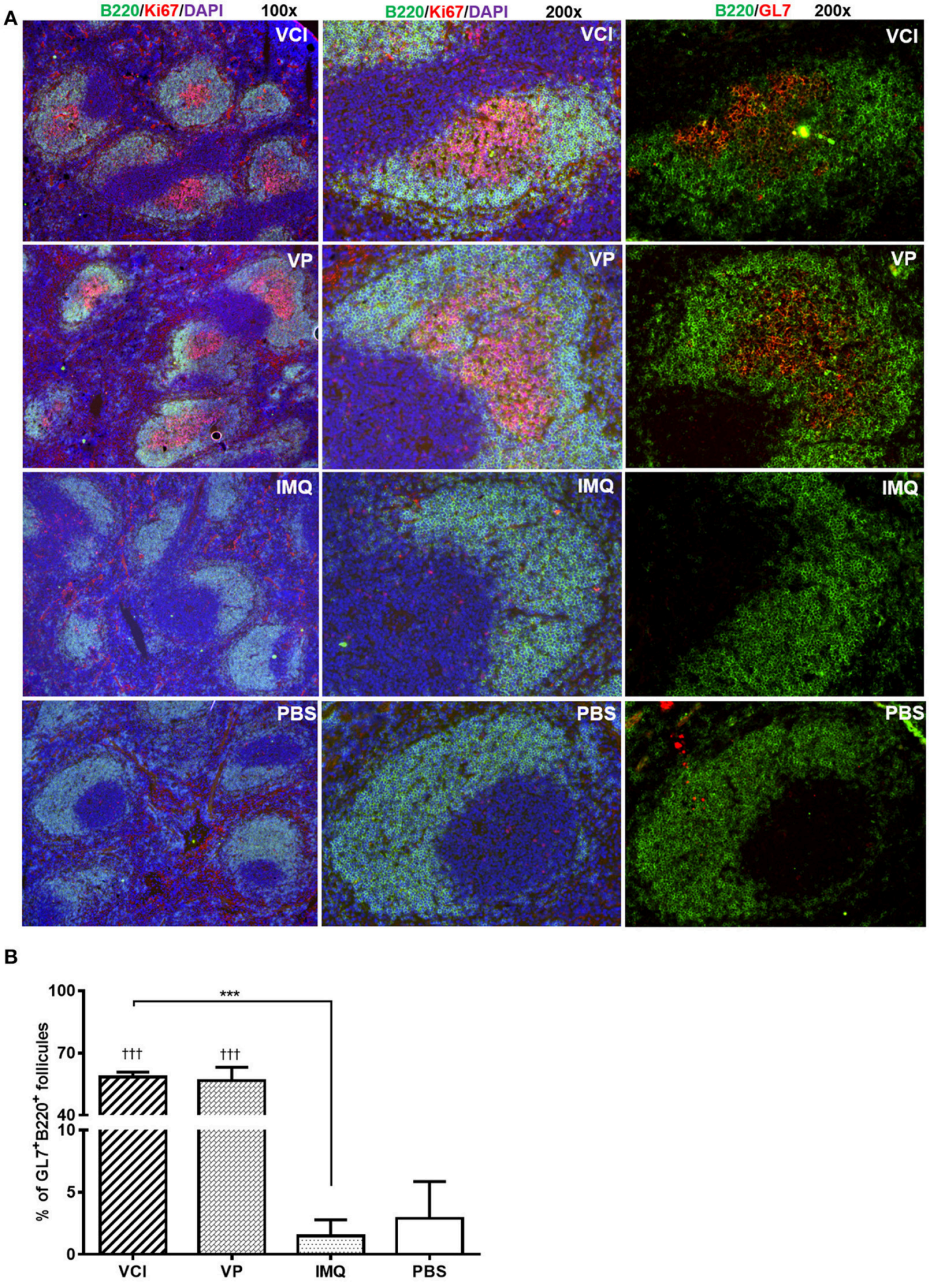
Immunofluorescent staining of mouse spleen for germinal center B cells. Mice received intraperitoneal VCI (IMQ 50 μg + VP 10 μg), IMQ (50 μg), VP (10 μg), or PBS for 3 days, and then were challenged with 10 × LD_50_ of H1N1/415742Md virus intranasally. At 4 days post virus challenge, spleens were taken and processed as frozen tissue sections. Consecutive sections were stained with anti-mouse CD45R/B220 and anti-mouse Ki67 or anti-mouse CD45R/B220 and anti-mouse GL7 antibodies. The stained tissues were examined under fluorescent microscope Olympus BX53. B cell follicles expressing B220/Ki67, B220/GL7 were counted and calculated. **(A)** Representative images of spleens stained with CD45R/B220 (green), Ki67 (red), GL7 (red) and DAPI (blue). Original magnification 100 × and 200 × . **(B)** Percentage of B220^+^/GL7+ B cell follicles in spleen at 4 days post virus challenge *n* = 3. ^†^^†^^†^*p* < 0.001 (compared with PBS group). ^***^p < 0.001; (comparing VCI group with IMQ group).

Then, using GL7 as a marker for B cell differentiation in germinal center response, we detected significantly more GL7 expressing B cells in the mdLN of the mice immunized with VCI than that treated with VP, IMQ, or PBS (Figures 6D,E); while the increase of GL7 expressing B cells in VCI immunized spleen was not significant comparing with VP or IMQ immunized mice. Using B220^+^/CD138^+^ as indicator for plasmablast cell and B220^lo^/CD138^+^ as marker for plasma cell differentiation (Figure 6F), we found significantly higher number of plasma cells in mdLNs of VCI-immunized mice, which are statistically significant compared to IMQ, VP alone (Figure 6G). To visualize the possible GC response in mouse spleen, frozen sections of spleen collected at 4 days post virus challenge were stained with B cell marker CD45R/B220, cell proliferation marker Ki67 and GC B cell marker GL7. As shown in Figure 7A, abundant B cell follicles showing double positive of B220 and proliferation marker Ki67 were observed in VCI and VP immunized mouse spleenspleens, further the results of GL7 staining showed that these B cells also expressing higher level of GL7. Semi-quantitative analysis showed that VCI and VP immunized mice had similar percentage of B cell follicles expressing Ki67 and GL7 (Figure 7B). This indicated that VCI and VP immunization both induced the GC formation in mouse spleens.

**Figure 8:**
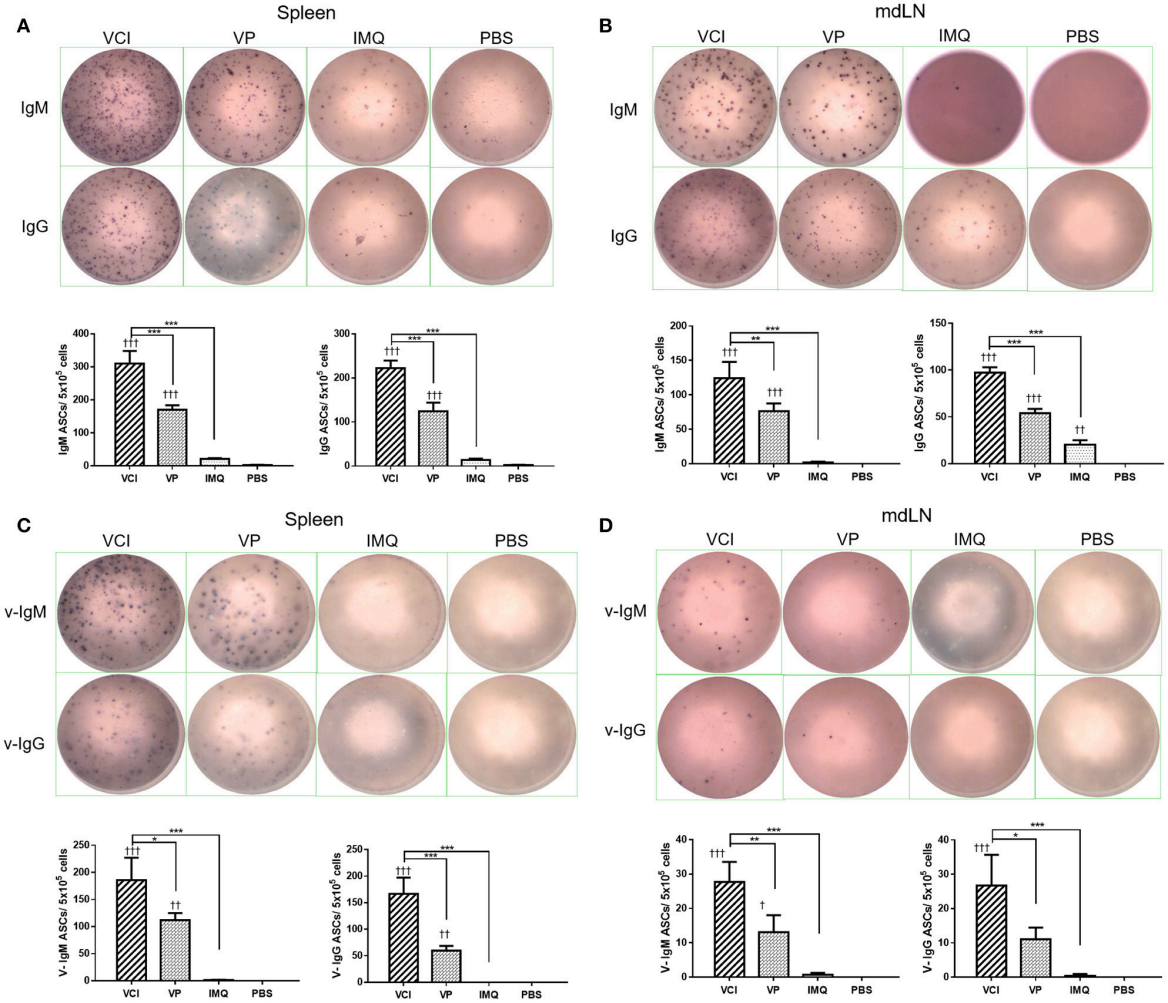
Antibody responses in spleen and mdLN. Mice received intraperitoneal administration of VCI (IMQ 50 μg + VP 10 μg), IMQ (50 μg) or VP (10 μg) or PBS for 3 days, and then were challenged with 10 × LD_50_ of H1N1/415742Md virus. At 3 days after virus challenge, single cells were prepared from spleens and mdLNs. ELISPOT detection of total IgM, IgG, viral specific IgM and viral specific IgG. **(A)** Total IgM, IgG secreting cells from spleen; **(B)** Total IgM, IgG spots from mdLN. **(C)** Viral specific IgM, IgG secreting cells from spleen; **(D)** Viral specific IgM, IgG secreting cells from mdLN. Data presented are mean of three different experiments for each type of the antibody secreting cells. *n* = 3. ^†^*p* < 0.05; ^†^^†^*p* < 0.01; ^†^^†^^†^*p* < 0.001 (compared with PBS group). ^*^p < 0.05; ^**^p < 0.01; ^***^p < 0.001 (comparing between VCI group with other treatment group).

Next, we determined whether there are any differences in the induction of ASC between VCI and other groups. At 3 days post virus challenge, single cell suspensions of mouse spleen and mdLN were tested for IgM and IgG secreting cells. The ELISPOT assay results showed that VCI immunized mice induced significantly higher number of IgM, IgG secreting cells in the spleen and mdLN comparing with that of VP or IMQ immunized mice (Figures 8A,B). But VP alone also induced significantly more IgM and IgG secreting cells comparing with PBS or IMQ treated mice. However, most importantly, viral specific IgM and IgG secreting cells significantly increased in the spleen and mdLN of VCI immunized mice comparing with other groups. VP group also had substantial number of viral specific IgM and IgG spots compared with those of IMQ or PBS immunized mice (Figures 8C,D). These data indicated that the antibody producing cells already exist in VCI immunized mice at 3 days post virus challenge, and to a less degree in VP immunized mice.

### *In vivo* administration of VCI elicited rapid antibody production in lung and serum and protected the mice from immediate lethal dose virus challenge

**Figure 9:**
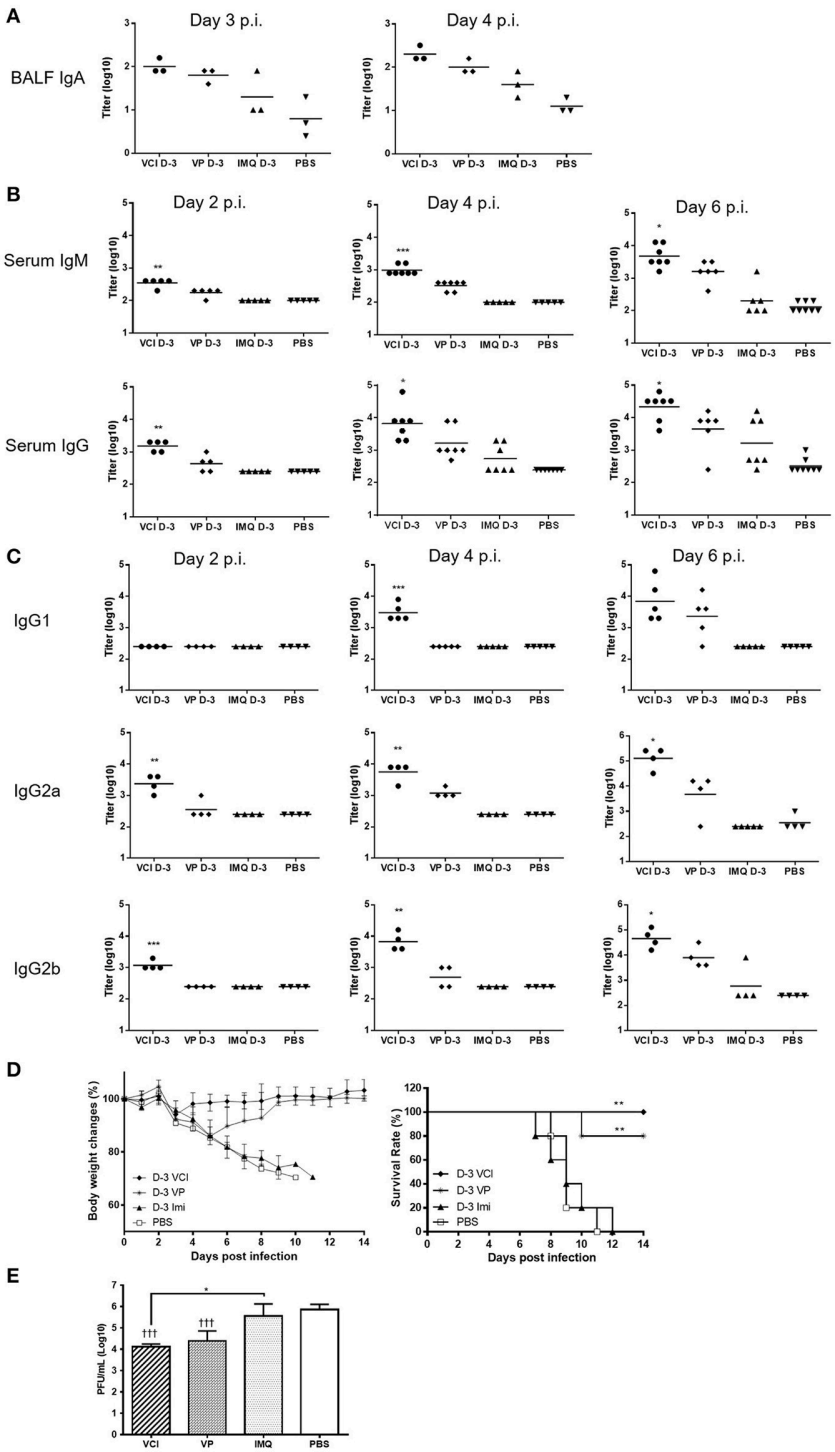
Lung and serum antibody responses in mice after H1N1/415742Md virus challenge. Mouse received intraperitoneal administration of VCI (IMQ 50 μg + VP 10 μg), IMQ (50 μg) or VP (10 μg) or PBS for 3 days, and then were challenged with 10 × LD_50_ of H1N1/415742Md virus. At day 2, 3, 4 or 6 days post virus challenge, BALF and serum was collected and tested by ELISA using inactivated H1N1/415742Md virus particles coated plates. **(A)** Viral specific IgA in BALF; **(B)** serum viral specific IgM, IgG; and **(C)** serum viral specific IgG1, IgG2a, IgG2b subtypes. The data are presented as titers on a log10 scale The horizontal lines indicate the geometric mean titers. Error bar indicates standard error of the mean. *n* = 3–8. ^*^p < 0.05; ^**^p < 0.01; ^***^p < 0.001 (comparing between VCI group with other treatment groups). **(D)** Body weight change (left) and survival rate (right) of mice after challenging with 10 × LD_50_ of H1N1/415742Md virus. *n* = 5. ^**^p < 0.01, when comparing with PBS group. **(E)** Lung viral load determined at 4 days post virus challenge. Error bar indicates standard deviation. *n* = 3, ^†^^†^^†^*p* < 0.001 (compared with PBS group), ^*^p < 0.05, when comparing with PBS group.

Determination of antibody production in bronchoalveolar lavage fluid by ELISA assay showed significantly higher level of viral specific IgA in BALF of VCI immunized mice at 3 and 4 days post virus challenge when compared with PBS control. VP immunization also induced higher level of viral specific IgA compared with PBS control, but was lower than that of VCI immunized mice (Figure 9A). IMQ treatment did not induce much viral specific IgA production compared to PBS control.

Mouse sera taken at 2, 4, and 6 days post virus challenge were tested for viral specific IgM and IgG. VCI immunization already elicited higher levels of viral specific IgM and IgG than other groups at 2 days post virus challenge (Figure 9B). The serum v-IgM and v-IgG titers increased at 4 and 6 days post virus challenge. VP alone induced production of IgM and IgG only on 4 and 6 post virus challenge, which was 2 days later than that of VCI immunized mice, and the v-IgM and v-IgG titers were only significantly increased at 6 days post virus challenge. For IgG subtypes, there was no significant difference in the titer of v-IgG1 among different treatment groups at 2 days post virus challenge, but the levels of v-IgG2a and v-IgG2b at 2 days post virus challenge were already reached significantly higher level in the VCI treated mice than others (Figure 9C), while VP alone only induced higher v-IgG2a and 2b at 6 days post virus challenge, which were still significantly lower than that of VCI.

To further show the protection effect of VCI immunization, mice challenged with 10 × LD50 H1N1/415742 virus were monitored for 14 days. VCI immunization group showed < 10% body weight loss at 3 days post challenge (Figure 9D, left), then regained weight and recovered with 100% survival (Figure 9D, right). VP immunized mice showed about 15% weight loss until day 5 and started to recover from day 6 post challenge with a 80% survival (Figure 9D). Mice in IMQ and PBS groups had severe body weight loss with 100% mortality (Figure 9D). VCI group had significant reduced lung viral load when compared with IMQ or PBS group, and was lower than VP group though not statistically significant (Figure 9E).

## Discussion

TLR7 agonist imiquimod has been shown to improve the immunogenicity of influenza virus vaccine in mice and in human (6–8), but the exact mechanism has not been fully understood. We specifically studied the effect of imiquimod on B cells in the presence of inactivated influenza virus (VP) as vaccine antigen. We demonstrated that the combination of imiquimod and inactivated virus (VCI) induced much stronger mouse peritoneal B cell activation and differentiation, when compared with VP or IMQ alone. We also demonstrated that peritoneal B cells stimulated with VCI *in vitro* secreted viral neutralizing antibody. Further, intraperitoneal administration of VCI conferred rapid B cell activation and differentiation in the spleen and lymph nodes. These B cells were capable to develop into antibody producing cells when encountered the virus only 3 days later, resulting in reduced lung viral load and 100% survival from lethal dose challenge. Therefore, the beneficial effect of imiquimod on vaccine is at least partially attributed to the accelerated B cell response.

The actions of imiquimod on human and murine B cells have previously been studied. Tomai et al. has shown that imiquimod can activate purified murine spleen B cells and human peripheral blood B cells and induce cell proliferation. But they found that imiquimod was ineffective at increasing murine spleen B cell to produce IgM and IgG (23). Douagi et al. emphasized the role of dendritic cells on enhancing imiquimod stimulated human peripheral B cells to produce IgM and IgG (24). Both studies were performed in the absence of vaccine antigen. In our study, we demonstrated consistently with their reports that imiquimod directly acted on murine peritoneal B cells to induce activation, proliferation and differentiation. From that, we extended to examine the effect of imiquimod in the presence of inactivated influenza virus. The results showed that this combination induced much stronger B cell responses to proliferate and differentiate into antigen specific IgM and IgG secreting cells which secreted antibodies with viral neutralizing activity. We therefore postulate that dual activation of TLR7 and B cell receptor (BCR) by imiquimod and VP are likely to be the underlying mechanism. In support of this, the cytokine responses from stimulated peritoneal B cells showed the IMQ or VP alone differentially upregulated cytokines expressions in which imiquimod induced stronger IL-10, while VP induced stronger inflammatory cytokine (IL-6), stronger Th-1 (IFN-γ), and Th-2 (IL-2, IL-4 and IL-5) cytokine responses. VCI combination upregulated both Th-1, Th-2 cytokines and IL-10. This suggested that IMQ and VP may activate different B cell signaling pathways leading to enhanced B cell responses to VCI. Furthermore, VCI stimulation upregulated the expression Blimp-1 and AICDA genes in peritoneal B cells which could contribute to the maturation and antibody production, but VP alone did not affect the expression of these two genes. Imiquimod alone induced peritoneal B cells activation, proliferation and differentiation to some degree but with little functional antibody; whereas VP by itself could only effectively activate B cells in the presence of other peritoneal cells. These results suggested that the TLR ligands presented in inactivated virus may not be readily accessible by TLR7 on purified B cells (25, 26), therefore co-stimulation signal from other immune cells, such as macrophages and dendritic cells, is needed to facilitate full activation of B cells. Another indirect evidence support dual-activation of TLR7 and BCR is that we found VCI stimulated B cell has much less apoptosis when compared with imiquimod or VP alone. Though it is currently unclear how VCI stimulation caused the reduction of apoptosis, previous studies have demonstrated that co-stimulation of TLR and BCR results in less apoptotic cell death in B cells than BCR or TLR stimulation alone (27).

The majority of the studies that evaluate adjuvant effects of imiquimod to vaccines were performed in conventional approaches which study vaccine antigen specific humoral or cellular immune responses against vaccines at 14, 28 days or even longer time period after immunization (28–30). The mechanisms of imiquimod were attributed to i) enhancing the activation and function of antigen presenting cells such as DC (31, 32), ii) inducing recruitment of CD8+ T cells (32–36) and iii) promoting B cell secretion of antibody (5, 23). Recently, Castiblanco et al. studied the effect of imiquimod on B cell response toward a hapten-protein antigen NP-CGG, and showed that imiquimod adjuvant improved memory B cell responses to the antigen (37). Our study differs from these reported studies. We aimed to study how quick a protective effect can be induced after VCI immunization. Our mice experiment showed that after intraperitoneal injection of VCI, the B cells in the spleen and distant lymph node were rapidly activated and differentiated with quick responsiveness to antigen re-stimulation 3 days after injection. At this stage, we do not know the origin of the increased B cells in spleen and lymph nodes, because we do not have direct evidence for the dynamic movement of peritoneal B cells upon VCI immunization. However, these splenic B cells are responsible to produce antigen-specific antibodies. This notion is supported by following the data: (1) transfer of VCI immunized spleen B cells conferred protection against homologous virus challenge in recipient naïve mice; (2) the serum of VCI immunized mice had viral neutralizing antibody only 3 days after immunization; (3) when those immunized mice were challenged with lethal dose influenza virus only 3 days after VCI immunization, they had significantly reduced lung viral load and 100% survival. Our previous study showed that 2-doses of IMQ combined with influenza HA2 recombinant protein antigen elicited significantly higher antibody responses than 2-does HA2 antigen alone groups (6), so we assume that boosting with second dose of antigen would augment the protective immunity. Imiquimod as vaccine adjuvant increasing the longevity of immune responses to vaccine has been reported. Our previous clinical study showed that combination of topical application of imiquimod with trivalent influenza vaccine induced sustainable higher titer of HAI and MN antibody which lasted 1 year after immunization (8). Castiblanco et al. studied reported that imiquimod as adjuvant improved memory B cell responses to hapten-protein antigen (37). However, whether VCI induced antibody responses could provide long term protection; whether this rapid immune response could provide protection against other influenza virus strains need further study.

Analysis of IgG subclasses showed that VCI immunized mice had early production of serum v-IgG2a and v-IgG2b at 2 days post virus challenges while IgG1 appeared at 4 days post challenge. Influenza vaccines have been showed in many reports that typically induced Th-2 type response, i.e., induce IgG1 antibody in BALB/c mice (38, 39). This suggested VCI induced rapid Th-1 antibody response. This finding is consistent with the previous report that TLR9 or TLR7 ligand coupled to virus-like particles shifted the antibody production to IgG2a, IgG2b, and IgG3 (40). IgG2a antibody response has been associated with increased influenza vaccination (41, 42). Both IgG1 and IgG2a contributed to the protection against lethal influenza virus challenge (42). Mouse Ig2a, IgG2b, and IgG1 are considered equivalent to human IgG1, IgG3 and IgG4, respectively (43), the major subclass of IgG detected in human serum after influenza infection or vaccination is IgG1 and then IgG2 or IgG3 (44–46). The results may provide some explanations to the early protective effect in mice challenged with influenza virus both in the current study and our previously reported monovalent pandemic H1N1 vaccine study (6). However, which subclass of IgG induced by VCI immunization provide most protection in our virus challenged mice is unclear. Combination of imiquimod and vaccine may induce antibodies with broader spectrum antigen, as our previous clinical study showed that intradermal vaccination combined with topical imiquimod increased antibody responses against non-vaccine strain and antigenic drifted strains (8). But whether VCI induced broadly neutralizing antibody against HA stem region which may provide protection against vaccine related stains, or even other subtype of influenza virus, warrant further studies. Further mapping the antibody responses would help to understand the mechanisms for VCI induced immunity.

We have chosen peritoneal B cells as a model for several reasons. First, our previous human vaccine trial was intradermal vaccine with topical imiquimod (8). The composition of peritoneal B cell population is similar to that of dermal B cell population. Innate B1 cell is the predominant B cell subtype in both the peritoneum and the dermal layer of the skin (47). Second, a large amount of B cells can be isolated from the peritoneum, while much less B cells can be isolated from the dermal layer. Study the response of peritoneal B cells to imiquimod and vaccine may advance our understanding of the mechanisms of intradermal vaccination with topical imiquimod treatment. There is limited information on the skin innate like B cell expression of TLRs or responses to TLRs activation. Geherin et al. reported that peritoneal B1 cell migrated to skin after LPS stimulation, where the B1 cells function as cutaneous immune sensory cells (48). This information also suggested that skin B1 cells may express TLR4 and response to TLR4 activation. Whether skin B1 cells directly response to topical TLR7 ligand imiquimod needs further study. One limitation of the current study is that peritoneal B cells were studied as a whole population without differentiating different subsets of B cells, for example B1 cell and B2 cell. The expression levels and responsiveness of TLRs in human or murine B cells vary depending on the B cell subtypes and activation status (49).To induce optimal regular B2 cells activation and maturation into antigen specific antibody producing cell requires TLR signaling and BCR signaling (50). *In vitro* stimulation of marginal zone (MZ) B cells with TLR4 ligand LPS induced significantly proliferation but no antibody secreting cell differentiation (51). Stimulation of peritoneal B1 cells with TLR2, TLR4, or TLR7/8 resulted in rapid induction of Blimp-1 gene and strong induction of IgM secretion. TLR9 activation also induced B1 cells into terminal differentiation (52). Therefore, it is reasonable to speculate that both innate B1 and regular B2 cells participated in viral specific antibody response induced by VCI *in vitro* and *in vivo*. Further study is needed to elucidate the differential effects of VCI on different B cell subsets.

Although VCI directly activated peritoneal B cells *in vitro* without the help from other cell types. The relative contributions of other cell, such as T helper cells, macrophages and dendritic cells to the *in vivo* effect induced by VCI has not been determined in our study. The peritoneal macrophages could be activated by VCI, and then could act on B cells by secreting cytokines or by presenting antigen to B cells. T cells have been shown to be activated by TLR2, 3, 4, 5, 7, and 8 agonist *in vitro* and *in vivo* both in human and in murine model (53). Different effects were observed in different subtypes of T cells. Funderburg et al. reported that imiquimod could only mildly activated human CD4^+^ and CD8^+^ T cells to proliferate *in vitro* (54). It is now perceived that T cells, especially Th-1 effector cells can be activated through direct action of microbial antigen on TLRs in the absence of antigen presenting cells (55). In this study, we showed significantly increased of CD3^+^CD4^+^ and CD3^+^CD8^+^ T cells in VCI immunized mouse spleens and mdLNs, which suggested the involvement of T cells. In addition, GC B cell proliferation and GL7 expression in VCI and VP immunized mouse spleens also suggested T cells involvement in the immune responses because GC development relies on T cell help. All these findings suggested that T cells are very likely to be activated in this experimental setting. However, how T cells were activated is unclear. B1 cells has been shown to express antigen presenting and co-stimulatory molecules including MHC class II, CD80, and CD86, indicating they play a role as antigen presenting cells (48, 56). Whether VCI activated peritoneal B cells could act as antigen presenting cell to prime T cells also needs further investigation.

In conclusion, our findings suggested TLR7 agonist imiquimod combined with vaccine antigen can promote potent B cell activation and differentiation leading to accelerated viral specific antibody production, which contribute to the protection against immediate incoming pathogen. This novel finding may perhaps be of clinical importance in the development of vaccination strategies and will shorten the immune-responsive time for early protection against epidemic pathogens.

## Ethics statement

Animal protocols were reviewed and approved by the Committee on the Use of Live Animals in Teaching and Research, the University of Hong Kong (CULATR #3726-15).

## Author contributions

CL performed large parts of the research and data analysis, and wrote the manuscript. KT was involved in the project design and writing the manuscript. AZ designed the experiments, wrote the manuscript, and supervised the experiments. AL performed part of flow cytometry assays and assisted the mouse experiments. HZ performed influenza virus inactivation and purification. WM did serum antibody ELISA assay and spleen germinal center staining. IH was involved in the project design. K-YY designed the project, was involved in writing the manuscript and supervised the whole project.

### Conflict of interest statement

The authors declare that the research was conducted in the absence of any commercial or financial relationships that could be construed as a potential conflict of interest.

## Supplementary material

Supplementary Figure S1 Representative ELISPOT images of antibody producing B cells after *in vitro* stimulation. Purified mouse peritoneal B cells were cultured in RPMI 1640 complete medium containing 2 μg/mL IMQ + 2 μg/mL VP (VCI), 2 μg/mL VP, 2 μg/mL IMQ, 2 μg/mL LPS, or medium only (CTL). Five or 7 days after stimulation, the cells were transferred to ELISPOT assay plates and further incubated for 24 h for detection of IgM or IgG secreting cells. **(A)** Representative ELISPOT images of total IgM (upper two panels) or IgG (lower two panels) secreting cells. **(B)** Representative ELISPOT images of viral specific IgM (upper two panels) or IgG (lower two panels) secreting cells.

Supplementary Figure S2 Representative images of immunofluorescent antibody stained viral NP antigen in FFMN assay. Purified mouse peritoneal B cells were cultured in RPMI 1640 complete medium containing 2 μg/mL IMQ + 2 μg/mL VP (VCI), 2 μg/mL VP, 2 μg/mL IMQ, 2 μg/mL LPS, or medium only (CTL). Seven days after stimulation, the culture supernatant were serial diluted and detected for FFMN assay to show peritoneal B cell culture supernatant neutralizing H1N1/415742Md virus infection of MDCK cells. NP positive cells were stained green, with DAPI stained nuclear. Original magnification 200 × .

Supplementary Figure S3 Representative flow cytometry profile of *in vitro* activation of mouse peritoneal B cells by IMQ and VP. Mouse whole peritoneal cells were cultured in RPMI1640 complete medium with or without IMQ or VP for 24 h. The cells were stained with FITC-CD19 and PE-CD86. Representative flow cytometric profiles after 24 h culture of cells stimulated with IMQ **(A)** or VP **(B)** (gated on live singlet).

Supplementary Figure S4 Representative images of functional antibody in serum of mice immunized for 3 days. Mice received intraperitoneal administration of VCI (IMQ 50 μg + VP 10 μg), IMQ (50 μg), VP (10 μg), or PBS. **(A)** Representative images of plaque inhibition by diluted mouse serum collected at 3 days after immunization. **(B)** Representative images of immunofluorescent antibody stained viral NP antigen in FFMN assay to show mouse serum neutralizing H1N1/415742Md virus infection of MDCK cells. NP positive cells were stained green, with DAPI stained nuclear. Original magnification 100 × .
