# Corrigendum: Co-stimulation with TLR7 agonist imiquimod and inactivated Influenza virus particles promotes mouse B cell activation, differentiation, and accelerated antigen specific antibody production

**DOI:** 10.3389/fimmu.2022.1053779

**Published:** 2022-10-21

**Authors:** Can Li, Kelvin K.W. To, Anna J. X. Zhang, Andrew C.Y. Lee, Houshun Zhu, Winger W.N. Mak, Ivan F. N. Hung, Kwok-Yung Yuen

**Affiliations:** ^1^ Department of Microbiology, Li Ka Shing Faculty of Medicine, University of Hong Kong, Pokfulam, Hong Kong; ^2^ State Key Laboratory for Emerging Infectious Diseases, University of Hong Kong, Pokfulam, Hong Kong; ^3^ Carol Yu Centre for Infection, University of Hong Kong, Pokfulam, Hong Kong; ^4^ Research Centre of Infection and Immunology, University of Hong Kong, Pokfulam, Hong Kong; ^5^ Department of Medicine, Li Ka Shing Faculty of Medicine, University of Hong Kong, Pokfulam, Hong Kong

**Keywords:** TLR7, imiquimod, inactivated influenza, A(H1N1)pdm09, B cell, peritoneal, mouse

In the published article, there were errors in **Figure 8** as published. The images of ELISPOT for PBS control treatment in Figure 8B, C and D were duplicated. The image of mdLN IgG ELISPOT in IMQ group (**Figure 8B**) was duplicated with spleen IgM in IMQ group (**Figure 8A**). The corrected Figure 8 and its caption appear below.

**Figure 8:**
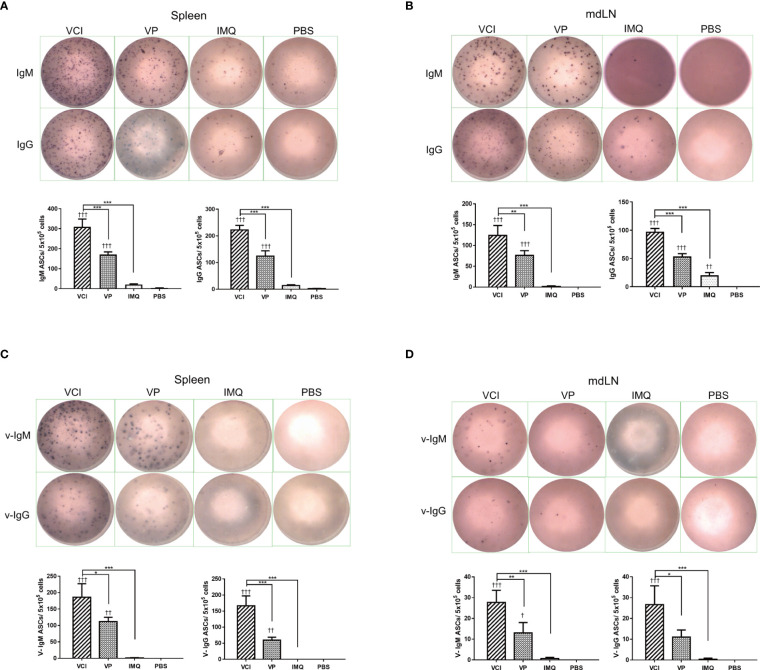
Antibody responses in spleen and mdLN. Mice received intraperitoneal administration of VCI (IMQ 50 µg + VP 10 µg), IMQ (50 µg) or VP (10 µg) or PBS for 3 days, and then were challenged with 10×LD50 of H1N1/415742Md virus. At 3 days after virus challenge, single cells were prepared from spleens and mdLNs. ELISPOT detection of total IgM, IgG, viral specific IgM and viral specific IgG. **(A)** Total IgM, IgG secreting cells from spleen; **(B)** Total IgM, IgG spots from mdLN. **(C)** Viral specific IgM, IgG secreting cells from spleen; **(D)** Viral specific IgM, IgG secreting cells from mdLN. Data presented are mean of three different experiments for each type of the antibody secreting cells. n = 3. ^†^p < 0.05; ^††^p < 0.01; ^†††^p < 0.001 (compared with PBS group). *p < 0.05; **p < 0.01; ***p < 0.001 (comparing between VCI group with other treatment group).

The authors apologize for these errors and state that they do not change the scientific conclusions of the article in any way. The original article has been updated.

## Publisher’s note

All claims expressed in this article are solely those of the authors and do not necessarily represent those of their affiliated organizations, or those of the publisher, the editors and the reviewers. Any product that may be evaluated in this article, or claim that may be made by its manufacturer, is not guaranteed or endorsed by the publisher.

