# Supplementary figures and images for: Co-stimulation With TLR7 Agonist Imiquimod and Inactivated Influenza Virus Particles Promotes Mouse B Cell Activation, Differentiation, and Accelerated Antigen Specific Antibody Production
Source: Front Immunol. 2018 Oct 12;9:2370. doi: 10.3389/fimmu.2018.02370 (PMC6194170; doi:10.3389/fimmu.2018.02370)

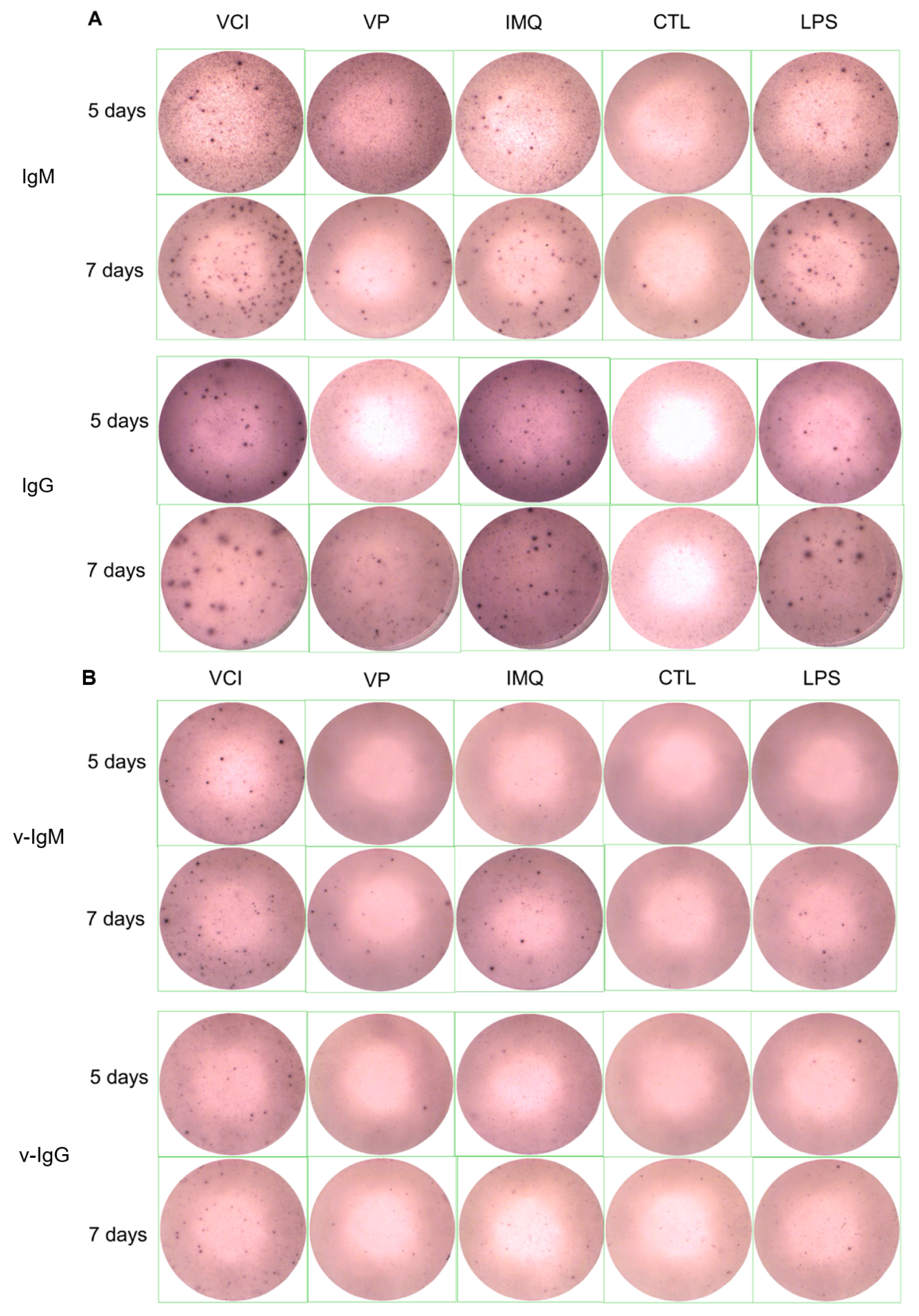

Supplement: Supplementary Figure S1 — Representative ELISPOT images of antibody producing B cells after in vitro stimulation. Purified mouse peritoneal B cells were cultured in RPMI 1640 complete medium containing 2 μg/mL IMQ + 2 μg/mL VP (VCI), 2 μg/mL VP, 2 μg/mL IMQ, 2 μg/mL LPS, or medium only (CTL). Five or 7 days after stimulation, the cells were transferred to ELISPOT assay plates and further incubated for 24 h for detection of IgM or IgG secreting cells. (A) Representative ELISPOT images of total IgM (upper two panels) or IgG (lower two panels) secreting cells. (B) Representative ELISPOT images of viral specific IgM (upper two panels) or IgG (lower two panels) secreting cells. [file Image_1.TIF]

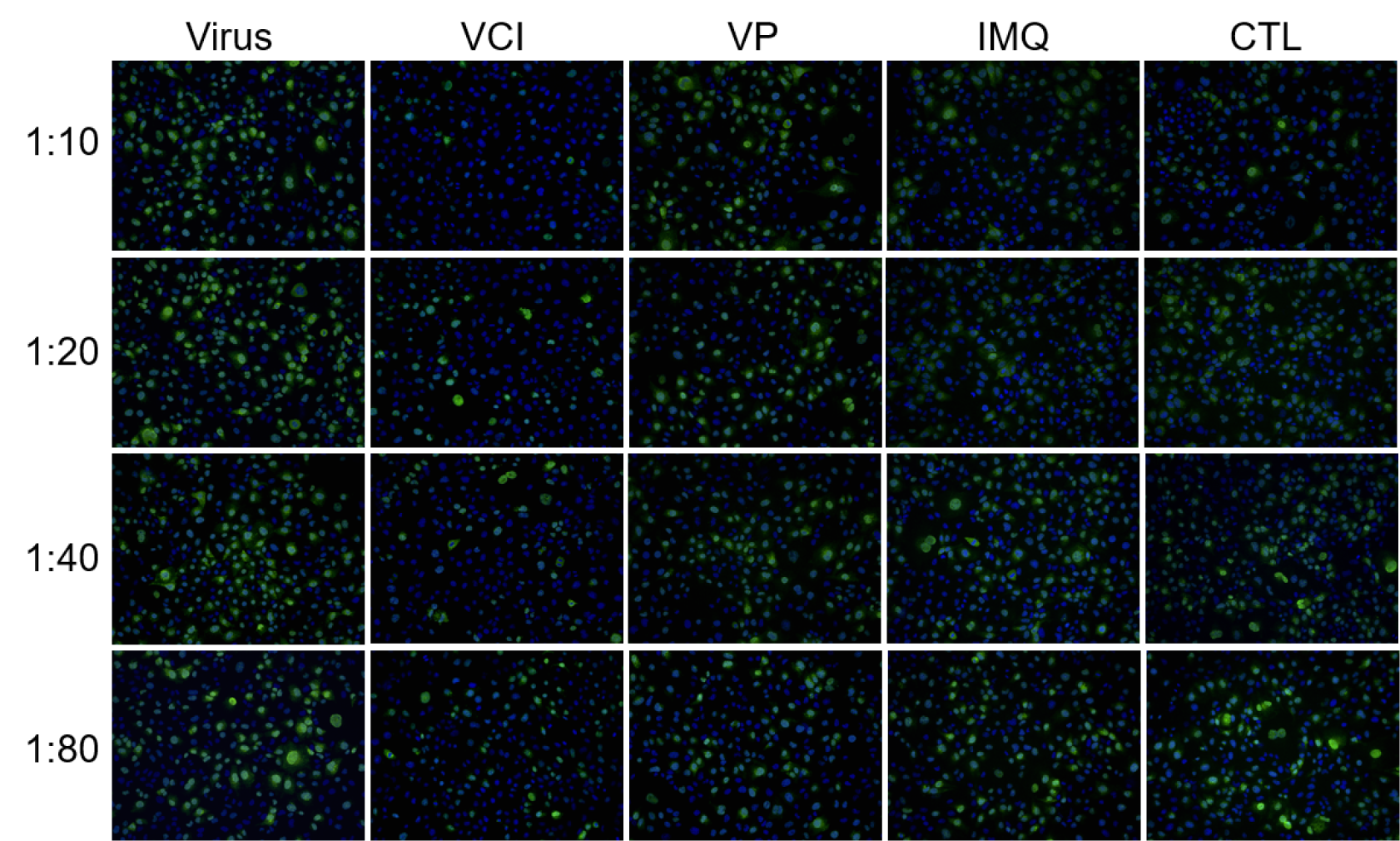

Supplement: Supplementary Figure S2 — Representative images of immunofluorescent antibody stained viral NP antigen in FFMN assay. Purified mouse peritoneal B cells were cultured in RPMI 1640 complete medium containing 2 μg/mL IMQ + 2 μg/mL VP (VCI), 2 μg/mL VP, 2 μg/mL IMQ, 2 μg/mL LPS, or medium only (CTL). Seven days after stimulation, the culture supernatant were serial diluted and detected for FFMN assay to show peritoneal B cell culture supernatant neutralizing H1N1/415742Md virus infection of MDCK cells. NP positive cells were stained green, with DAPI stained nuclear. Original magnification 200 × . [file Image_2.TIF]

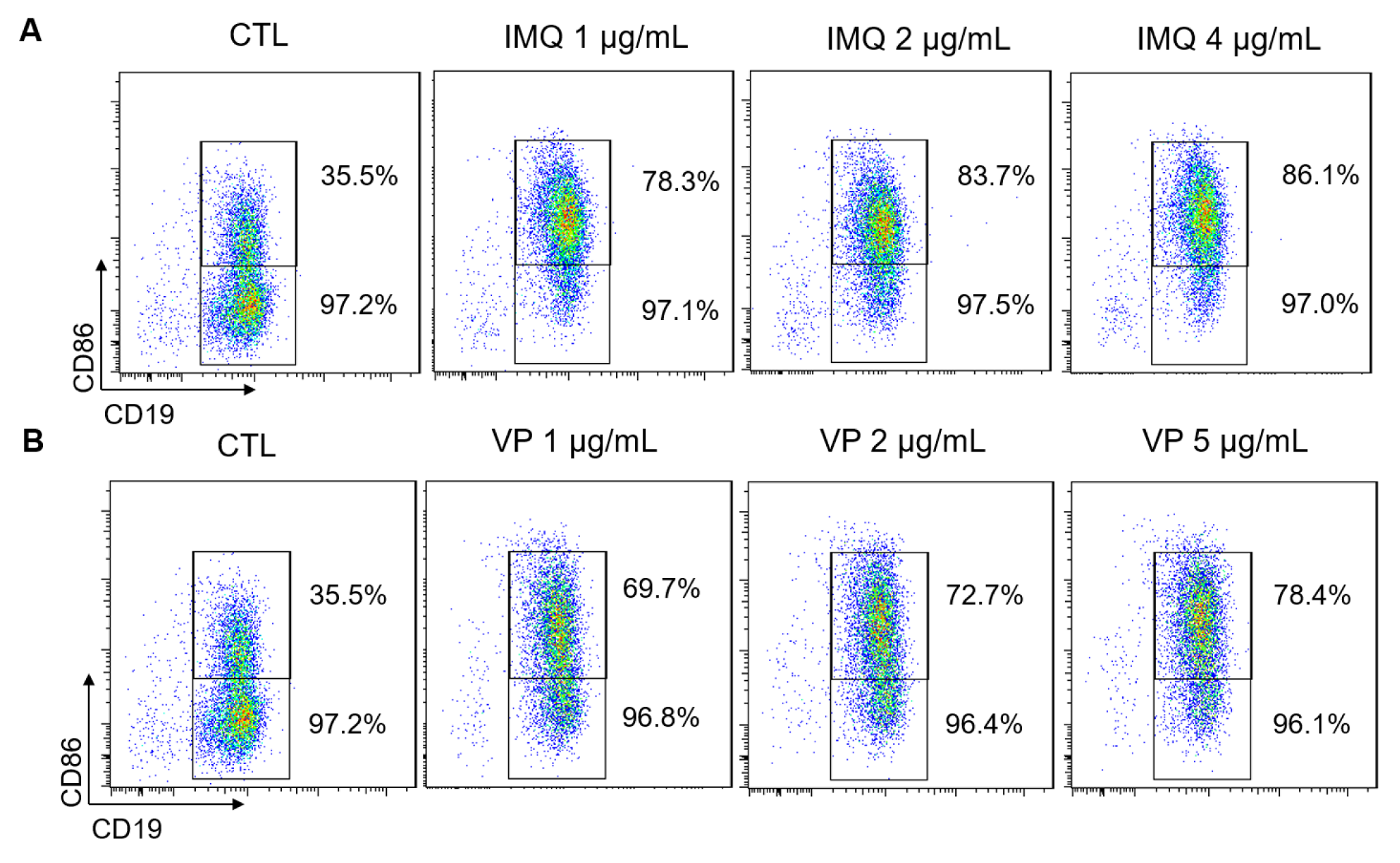

Supplement: Supplementary Figure S3 — Representative flow cytometry profile of in vitro activation of mouse peritoneal B cells by IMQ and VP. Mouse whole peritoneal cells were cultured in RPMI1640 complete medium with or without IMQ or VP for 24 h. The cells were stained with FITC-CD19 and PE-CD86. Representative flow cytometric profiles after 24 h culture of cells stimulated with IMQ (A) or VP (B) (gated on live singlet). [file Image_3.TIF]

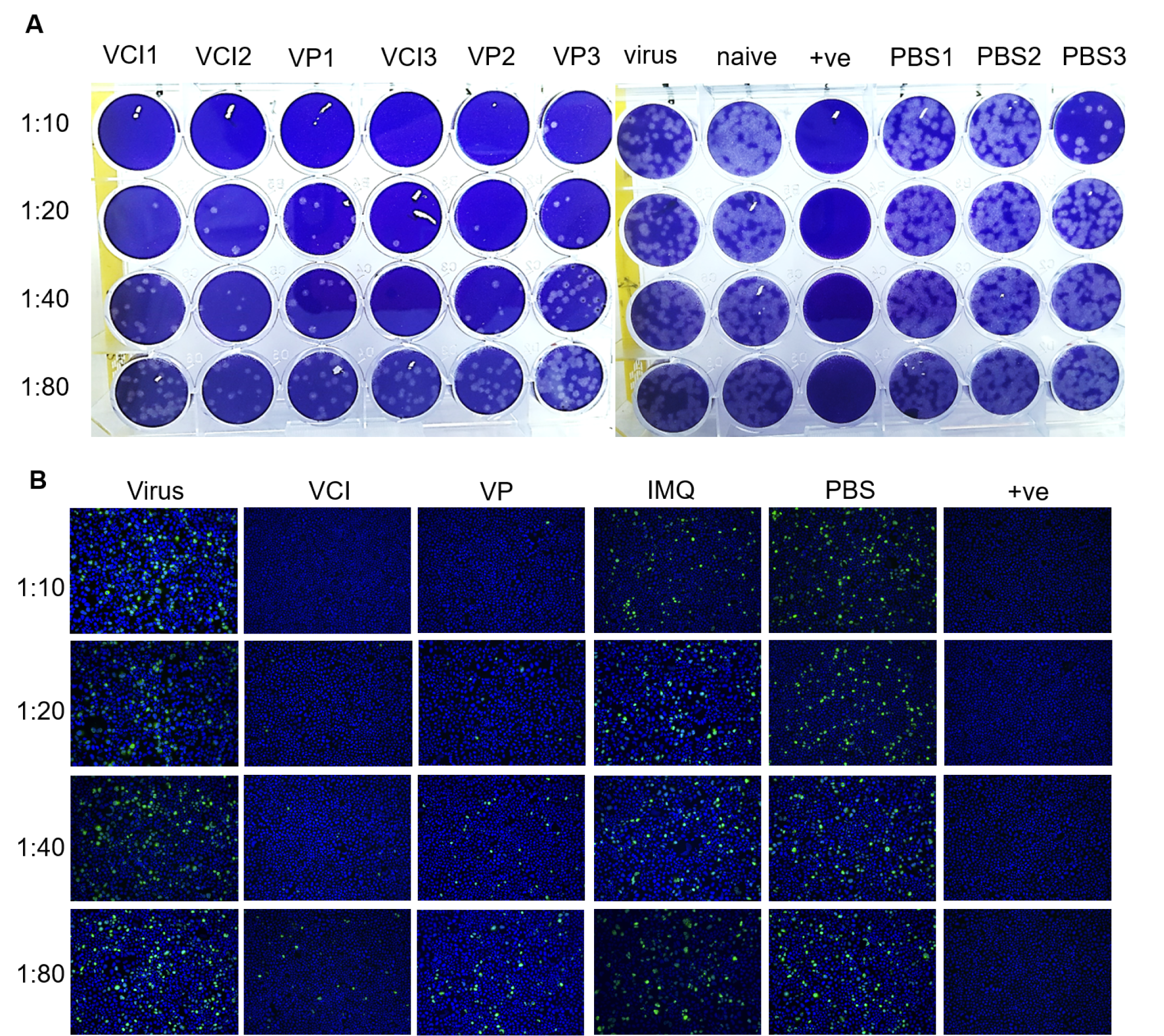

Supplement: Supplementary Figure S4 — Representative images of functional antibody in serum of mice immunized for 3 days. Mice received intraperitoneal administration of VCI (IMQ 50 μg + VP 10 μg), IMQ (50 μg), VP (10 μg), or PBS. (A) Representative images of plaque inhibition by diluted mouse serum collected at 3 days after immunization. (B) Representative images of immunofluorescent antibody stained viral NP antigen in FFMN assay to show mouse serum neutralizing H1N1/415742Md virus infection of MDCK cells. NP positive cells were stained green, with DAPI stained nuclear. Original magnification 100 × . [file Image_4.TIF]
